# Mind the gap: a systematic review of barriers, facilitators, and experiences of care transitions for people living with dementia and their informal caregivers

**DOI:** 10.1093/geront/gnaf275

**Published:** 2025-11-25

**Authors:** Leanne Greene, Jacob Brain, Paige Watkins, Aysegul Humeyra Kafadar, Serena Sabatini, Barbara Blundell, Ellen Bothe, Kristie Harper, Deborah Hersh, Claire Morrisby, Bronwyn Myers, Andrew Stafford, Eugene Y H Tang, Blossom C M Stephan, Elissa Burton

**Affiliations:** Curtin enAble Institute, Faculty of Health Sciences, Curtin University, Bentley, Perth, Western Australia, Australia; Institute of Mental Health, School of Medicine, University of Nottingham, Nottingham, United Kingdom; Freemasons Foundation Centre for Men’s Health, Discipline of Medicine, School of Psychology, The University of Adelaide, Adelaide, South Australia, Australia; Curtin enAble Institute, Faculty of Health Sciences, Curtin University, Bentley, Perth, Western Australia, Australia; Institute of Mental Health, School of Medicine, University of Nottingham, Nottingham, United Kingdom; Curtin enAble Institute, Faculty of Health Sciences, Curtin University, Bentley, Perth, Western Australia, Australia; Curtin enAble Institute, Faculty of Health Sciences, Curtin University, Bentley, Perth, Western Australia, Australia; Curtin School of Allied Health, Curtin University, Bentley, Perth, Western Australia, Australia; Curtin enAble Institute, Faculty of Health Sciences, Curtin University, Bentley, Perth, Western Australia, Australia; Curtin enAble Institute, Faculty of Health Sciences, Curtin University, Bentley, Perth, Western Australia, Australia; Curtin School of Allied Health, Curtin University, Bentley, Perth, Western Australia, Australia; Sir Charles Gairdner Osborne Park Health Care Group, Occupational Therapy, Nedlands, Perth, Western Australia, Australia; Curtin enAble Institute, Faculty of Health Sciences, Curtin University, Bentley, Perth, Western Australia, Australia; Curtin School of Allied Health, Curtin University, Bentley, Perth, Western Australia, Australia; School of Allied Health Science and Practice, University of Adelaide, Adelaide, South Australia, Australia; Curtin enAble Institute, Faculty of Health Sciences, Curtin University, Bentley, Perth, Western Australia, Australia; Curtin School of Allied Health, Curtin University, Bentley, Perth, Western Australia, Australia; Curtin enAble Institute, Faculty of Health Sciences, Curtin University, Bentley, Perth, Western Australia, Australia; West Australian Country Health Service (WACHS)-Curtin University Research and Innovation Alliance, Curtin University, Perth, Western Australia, Australia; Mental Health, Alcohol, Substance Use and Tobacco Research Unit, South African Medical Research Council, Tygerberg, South Africa; Curtin enAble Institute, Faculty of Health Sciences, Curtin University, Bentley, Perth, Western Australia, Australia; Curtin Medical School, Curtin University, Bentley, Perth, Western Australia, Australia; Population Health Sciences Institute, Newcastle University, Newcastle, United Kingdom; Curtin enAble Institute, Faculty of Health Sciences, Curtin University, Bentley, Perth, Western Australia, Australia; Curtin enAble Institute, Faculty of Health Sciences, Curtin University, Bentley, Perth, Western Australia, Australia; Curtin School of Allied Health, Curtin University, Bentley, Perth, Western Australia, Australia; Brightwater Research Centre, Brightwater Care Group, Inglewood, Perth, Western Australia, Australia

**Keywords:** Caregivers, Patient transfer, Patient discharge, Terminal care, Patient-centered care

## Abstract

**Background and Objectives:**

Care transitions for people living with dementia are critical periods requiring coordinated, person-centered support. Effective transitions can reduce caregiver burden, prevent adverse outcomes, and improve care quality. However, the barriers, facilitators, and lived experiences during transitions remain poorly understood. This systematic review synthesizes evidence on these factors from the perspectives of people with dementia and their informal caregivers.

**Research Design and Methods:**

A comprehensive search across MEDLINE, CINAHL, PsycINFO, ProQuest, and Web of Science identified 67 eligible English-language studies published from 2018 to 2023. Quality appraisal used Joanna Briggs Institute tools. The protocol was registered on PROSPERO: CRD42023452669.

**Results:**

Four themes captured the barriers, facilitators, and experiences shaping care transitions for people with dementia and their caregivers. Systemic influences included fragmented governance, funding and policy inconsistencies, and structural challenges in care coordination and delivery, mitigated by proactive planning and integrated care. Health and social care workforce factors highlighted gaps in dementia training, staffing, and communication, with empathetic, informed staff improving transitions. Emotions and decision making reflected caregiver burden, uncertainty, and advocacy, eased by early guidance and peer support. Cultural, social and situational influences showed how values, socioeconomic status, and rurality affected transition choices, underscoring the need for culturally sensitive, person-centered support.

**Discussion and Implications:**

Care transitions remain complex, shaped by systemic, workforce, emotional, and cultural factors. Addressing inequities and coordination gaps is critical for more integrated transitional care. Strengthening dementia-specific training, home-based care models, and culturally responsive communication may improve continuity, person-centeredness, and caregiver support.

## Background

Care transitions represent some of the most emotionally and logistically challenging experiences for people living with dementia and their informal caregivers (Ashbourne et al., 2021). Care transitions, defined as the anticipated movement of individuals between different levels or locations of care (Downs & Bowers, 2014), can include acute hospital admissions, emergency department visits, respite care episodes, moves from home to long-term care facilities, and transfers between hospital departments. Transitional periods represent particularly vulnerable moments for people with dementia and their caregivers due to the disruption of care continuity, communication challenges, heightened risk of adverse events, and the emotional and logistical burden placed on caregivers navigating complex health systems (Cronfalk et al., 2018; Delgado et al., 2022; Fogg et al., 2018).

The complexity of these transitions is amplified by the progressive nature of dementia, which is characterized by cognitive decline and behavioral changes, leading to increasingly complex care needs and a growing dependence on informal caregivers (Ashbourne et al., 2021). These care demands are often further complicated by comorbid conditions, requiring frequent interaction with multiple health care providers and settings. As a result, people living with dementia experience care transitions more often and more challenging than the general population (Callahan et al., 2015; Downs & Bowers, 2014; Phelan et al., 2012).

Despite the critical importance of coordinated care during these transitions, people with dementia often experience care fragmentation (Fogg et al., 2018; Martin et al., 2020; Smith et al., 2021). This fragmentation can manifest as poor information transfer between providers, inadequate discharge planning, insufficient support for family caregivers, and lack of follow-up care (Ashbourne et al., 2021; Saragosa et al., 2022). This fragmentation was particularly problematic for people with dementia who may have difficulty communicating their needs and medical history, increasing their risk of adverse outcomes during transitions (Kern et al., 2024; Naylor & ­Keating, 2008).

The challenges of care transitions in dementia are further complicated by the diverse needs of both the person with dementia and their caregivers. Family caregivers often play a crucial role in managing these transitions, yet many report feeling overwhelmed, underprepared, and inadequately supported during this process (Ashbourne et al., 2021). Health care providers similarly face challenges in coordinating care across settings, particularly when dealing with complex medical needs alongside cognitive impairment (Österholm et al., 2023).

Understanding the barriers, facilitators, and lived experiences of dementia-related care transitions is essential for developing effective interventions and policies to enhance the transition experience and outcomes. While previous research has examined aspects of care transitions for people with dementia and their caregivers, a systematic review that synthesizes evidence regarding the factors that influence transition outcomes has not been conducted. Given the projected increase in dementia prevalence and the resulting rise in care transitions, this systematic review seeks to offer a comprehensive understanding of what is known about the barriers, facilitators, and lived experiences of these transitions from the perspective of people with dementia and their informal caregivers. By synthesizing the current evidence, this review may inform future research directions, policy initiatives, and practical interventions to support more effective and compassionate care transitions for people with dementia and their caregivers.

Understanding how people living with dementia and their informal caregivers experience care transitions, and the barriers and facilitators they encounter, is essential for informing future research, policy, and practice. These perspectives provide critical insights into how transitions affect emotional well-being, autonomy, continuity of care, and overall quality of life. Informal caregivers also play a central role in coordinating care, advocating for their loved ones, and managing responsibilities after discharge, often with limited support. Including their voices offers a more holistic understanding of care transitions and is vital for designing more compassionate care approaches. With dementia currently affecting an estimated 57 million people globally and projected to reach 153 million by 2050 (Nichols et al., 2022), the need for person- and family-centered support is urgent (Long et al., 2023; World Health Organization, 2023).

Existing reviews have focused specifically on the development and use of standardized tools and metrics to evaluate different aspects of care transitions and coordination, rather than on descriptive accounts of transitions themselves (Hirschman & Hodgson, 2018; Hirschman et al., 2023), or outcomes related to particular transition points, such as the move from day respite services to permanent residential aged care (Meyer et al., 2024). To our knowledge, no systematic review has comprehensively synthesized the perspectives of both people living with dementia and their informal caregivers across a range of care transition types, while also examining the barriers, facilitators, and lived experiences they encounter. This broader synthesis offers a more holistic understanding of the care transition experience and addresses a critical gap in the literature by contextualizing lived experiences within the wider systemic and structural factors that shape these transitions.

## Research design and methods

### Design

Through preliminary scoping of the literature, we identified two complementary dimensions of care transitions that warrant systematic investigation: the experiential aspects (barriers, facilitators, and lived experiences) and the epidemiological aspects (frequency, timing, risk factors, and trajectories). This led to the development of a planned series of systematic reviews to examine this critical area of dementia care in depth.

The first review (Paper 1), presented here, synthesizes evidence on the barriers, facilitators, and lived experiences of care transitions from the perspectives of people living with dementia and their informal caregivers. The second planned review (Paper 2) will examine the epidemiological aspects of care transitions, including their frequency, timing, associated risk factors, and common patterns or trajectories among people living with dementia. To ensure consistency in the evidence base, the literature search for both reviews was conducted simultaneously, allowing for comprehensive data extraction from the same time point.

### Protocol and registration

This systematic review follows the recommendations of the Preferred Reporting Items for Systematic Reviews and Meta-Analyses Protocols statement (Page et al., 2021) (supplementary Appendix A) and was registered on the International Prospective Register of Systematic Reviews (PROSPERO: CRD42023452669; https://www.crd.york.ac.uk/prospero/display_record.php? RecordID=452669).

### Literature search

In collaboration with a Curtin University Librarian, five electronic databases, MEDLINE, CINAHL, PsycINFO, ProQuest (Health and Medicine), and Web of Science, were initially searched from January 1, 2000 to July 11, 2023 resulting in the retrieval of 28,229 articles. The following primary concepts were included in the search strategy and mapped to Subject Headings (e.g., MeSH) where appropriate: dementia, Alzheimer’s disease, transition, and aged care (supplementary Appendix B). In addition to the database searches, backwards citation searching was undertaken by reviewing the reference lists of all included studies. Titles were screened for relevance to the review topic, and abstracts and full texts were reviewed as necessary based on the predefined inclusion criteria; however, this process did not yield any additional eligible studies.

### Eligibility

Eligible studies were peer-reviewed publications using any primary research methodology, including randomized controlled trials (RCTs), cohort, case–control, quasi-experimental, cross-sectional, qualitative, and mixed-methods study designs. Studies were included if the population consisted of people with a clinical diagnosis of dementia (e.g., International Classification of Diseases [World Health Organzation, 1978] or Diagnostic and Statistical Manual of Mental Disorders [American Psychiatric Association, 2013]) across all dementia subtypes. Studies focusing solely on informal, unpaid caregivers of people with dementia were also eligible for inclusion. In these cases, the person with dementia was not a study participant, and formal diagnostic details were typically not reported. Studies were included as long as they explicitly identified participants as caregivers of individuals with dementia. The exposure involved any type of care transition, such as transitions between primary, secondary, tertiary, community, and home care settings. Examples of transitions include ambulance transfers, emergency department or hospital admissions, moving into long-term care (e.g., nursing homes), or respite care. Home and community care, even if delivered at home, were also classified as care transitions. To be included in this review, studies were required to report findings related to at least one of the following: barriers, facilitators, or lived experiences of care transitions. Barriers referred to factors that hindered care transitions, facilitators were those that supported or improved them, and lived experiences captured the personal perspectives of individuals navigating these transitions. Articles were only included if at least 50% of the population consisted of people with dementia or their caregivers. No restrictions were placed on the country of origin of included studies, as incorporating research from diverse international settings allowed for a more comprehensive synthesis of how varying health care systems and policy contexts shaped dementia-related care transitions.

Excluded studies included secondary research, such as systematic, scoping, and umbrella reviews, as well as meta-analyses, protocols, and case reports/series. Non-peer-reviewed publications, including dissertations, conference abstracts, letters to editors, and opinion pieces, were also excluded. Articles not published in English were excluded. In terms of population, exclusions applied to people living with mild cognitive impairment or subjective memory (or cognitive) problems, paid formal caregivers, and staff perspectives. No age restrictions were applied as care transitions across the dementia continuum, including early-onset and late-onset dementia, were of interest.

After full-text screening, we decided to limit the search to studies from the past 5 years (January 1, 2018–July 11, 2023) to ensure that evidence on care transitions was relevant to current dementia care practices. Since 2018, there have been advancements and policy changes in dementia care (e.g., National Dementia Strategies [North West Coast Strategic Clinical Network, 2018] and WHO’s Global Action Plan on the Public Health Response to Dementia [World Health Organization, 2020], EU4Health Programme [European Commission, 2021]).

### Study selection

Records identified through the initial search underwent de-duplication using the built-in functionalities of both EndNote (Clarivate, 2013) and Covidence (Veritas Health Innovation, 2014) software. Subsequently, four reviewers (L.G., J.B., S.S., A.H.K.) independently screened titles and abstracts against the eligibility criteria. Double screening was used, and any discrepancies in the initial screening were adjudicated by a third independent reviewer (E.Bu.), who made the final determination on study inclusion for full-text review. Each full text was screened by two independent reviewers (L.G., J.B., A.H.K., E.Bu., C.M., E.B., K.H., A.S., D.H., B.B., S.S., B.C.M.S., B.M.), with conflicts resolved through discussion (L.G., J.B., E.Bu.).

### Quality assessment

The quality of the included studies was assessed using the Joanna Briggs Institute (JBI) Critical Appraisal Toolkits. Given the diverse study designs included in this review, we applied the relevant JBI checklist based on study type (for qualitative research, cohort studies, quasi-experimental studies, RCTs, cross-sectional studies, and case–control studies). Each study was appraised by two independent authors (L.G., J.B., S.S., A.H.K., P.W., C.M., E.Y.H.T., K.H., A.S., E.Bu.), with disagreements resolved through discussion. No studies were excluded based on the quality appraisal. This decision was made a priori, as our aim was to synthesize the full body of available evidence relating to the experiences, barriers, and facilitators of care transitions.

### Data extraction

Data were independently extracted by three authors (L.G., J.B., P.W.) and cross-checked by two authors (L.G. and J.B.) using a predesigned data extraction sheet which was initially piloted by four authors (L.G., J.B., P.W., E.Bu.). The following data were extracted: author, year of publication, country of study, sample size, study design, dementia subtype, the experience of transition, facilitator of transition, barrier to transition, and variations in transitions. Data on facilitators, barriers, and experiences were extracted either when these concepts were explicitly labelled by the original study authors (e.g., “barriers,” “challenges,” “enablers,” or “facilitators”), or through interpretive analysis by the reviewers. When not explicitly named, reviewer judgment was applied to identify findings that clearly described positive or negative influences on care transitions, or that reflected lived experiences relevant to the review aims. Data were also extracted on variations in transitions where relevant, including, but not limited to, differences associated with dementia subtype, cultural context, age, systemic factors such as health care governance, policy frameworks, and the organization and structural factors like design of delivery and coordination of care within public *versus* private systems.

### Synthesis of evidence

A thematic synthesis approach was used to integrate findings from studies with diverse methodological designs, following three key stages (Thomas & Harden, 2008). Study findings were first coded line by line to capture meaning and context, allowing concepts to emerge inductively from the data. These codes were then organized into descriptive themes that reflected patterns across studies. Finally, analytical themes were developed by interpreting and integrating these patterns to generate higher-level insights that addressed the review aims and explained how barriers, facilitators, and lived experiences shape care transitions for people with dementia and their caregivers. Data from the extraction sheet were imported into NVivo (QSR International Pty Ltd., 2020), where the lead author (L.G.) conducted both inductive and deductive thematic analysis to code and categorize the data. Given the review’s focus on barriers, facilitators, and lived experiences of care transitions for people with dementia, these elements formed the initial deductive codes. Inductive coding was then applied to identify emerging subthemes. A framework matrix was developed within NVivo to organize and refine the data into thematic categories. All authors reviewed and agreed on the final themes.

## Results

**Figure 1.**
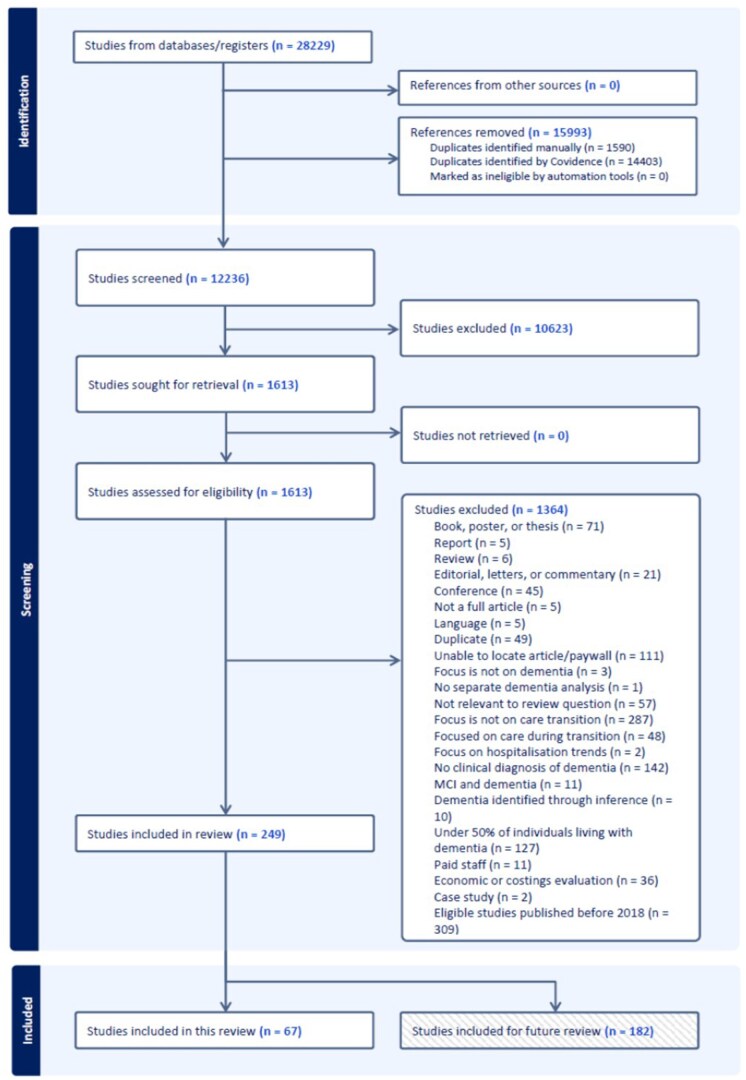
PRISMA diagram.

In total, 28,229 records were identified through the electronic search. After removing 15,993 duplicate records, 12,236 titles and abstracts were screened. Of these, 1,613 articles were selected for full-text review, and 560 studies met the initial inclusion criteria. Applying the date range of 2018–2023 reduced the number to 249 studies. These were then categorized by relevance: 67 studies were included in this review, which focused on barriers, facilitators, and experiences of care transitions; the remaining 182 studies will be included in a forthcoming review on epidemiological aspects of care transitions for people with dementia (Figure 1).

A total of 67 studies were included in this review, comprising qualitative studies (*n *= 31), cohort studies (*n *= 15), cross-sectional studies (*n *= 5), quasi-experimental studies (*n *= 4), RCTs (*n *= 4), case–control studies (*n *= 1), mixed-method studies combining qualitative and cohort designs (*n *= 2), qualitative and RCT designs (*n *= 3), and qualitative and cross-sectional designs (*n *= 2). Of the 67 studies, the majority were conducted in high-income, Western countries including the United States (*n *= 20), Australia (*n *= 15), Canada (*n *= 6), United Kingdom (*n *= 6), Netherlands (*n *= 3), Norway (*n *= 5), New Zealand (*n *= 2), Sweden (*n *= 2), Belgium (*n *= 1), France (*n *= 1), Germany (*n *= 1), Ireland (*n *= 1), and Slovenia (*n *= 1). Only three studies were conducted in Asia: Taiwan (*n *= 2) and China (*n *= 1). See Table 1 for study characteristics.

**Table 1.** Characteristics of included studies.

Number of study	Author and date	Country	Sample size	Study design	Described transition context	Experience of transition	Facilitators of transition/Factor reducing transitions	Barriers of transition
**1**	Armstrong 2020	United States	30	Qualitative	End of life, hospitals, SNF, hospice, home care	NR	Improved communication with facility staff and fewer barriers in home care services facilitated care transitions. Hospice care was positively experienced once accessed, whether at home or in facilities.	Misdiagnosis, delayed or conflicting diagnoses, and a lack of knowledge across health care systems created significant challenges. Poor care coordination and high costs added system-level barriers. Facilities faced issues like inadequate staffing, staff turnover, limited DLB expertise, poor communication, and family conflicts. Home care was hindered by difficulty finding qualified staff and insufficient caregiving support. Hospice presented challenges such as strict entry criteria, loss of physical therapy, poor coordination, staff turnover, and medication issues. Caregivers also faced insufficient physician knowledge, lack of education, and inadequate support.
**2**	Ashbourne 2021	Canada	29	Qualitative	NR, but included primary care providers, specialists, memory clinics, hospital, respite care, LTC home, and home care providers	Caregivers worry that the health care system did not have essential information on the person living with dementia. System constraints prioritized over their view of needs. Feeling loss of control which increased stress for caregiver. Not knowing what to do with their time now the person living with dementia is in LTC.	Supportive communities, shared caregiver experiences, gradual transitions, coordinated follow-ups, consistent providers, LTC familiarization, proactive preparation, compassionate health care providers, and joint decision making with person living with dementia.	Gradual transitions sometimes delayed addressing needs, system constraints overlooked caregiver priorities, complex health care systems were difficult to navigate, and providers lacked knowledge and clear information.
**3**	Broadbent 2020	Australia	11	Qualitative	NR to memory support unit (high-level dementia care)	NR	Specialized environments for people living with dementia, spacious and familiar building designs, and settings aligned with residents’ previous lifestyles eased transitions and reduced family worry.	NR
**4**	Brooks 2022b	Australia	21	Randomized-control trial	Home/hospital/other to RACF	Caregivers: intervention acceptable and useful. Feelings of guilt, loss and grief. Lower perceived stress, depression, and “nursing home hassles.” Greater “acceptance of loss”	Safe discussions with experienced dementia care professionals, free from judgment, and access to problem-solving and coping strategies.	NR
**5**	Brooks 2022a	Australia	9	Qualitative	Home to long-term care	Caregivers experienced significant stress and emotional strain, exacerbated by administrative challenges, pressure for quick placement decisions, and lack of choice or formal support following hospitalization. They faced losses of personal identity and caregiving control, feelings of loneliness and depression, and persistent grief. Limited or inconsistent support from family and friends intensified guilt. Stress was heightened by issues such as locked doors, understaffing, impersonal care, disruptive resident behaviors, and disputes with staff over care decisions. Caregivers also reported distress, anxiety, and mental exhaustion.	Care home brokers or placement consultants eased navigation and reduced stress. Respite care facilitated LTC transitions, and preplacement visits improved understanding. Educational support on dementia progression helped manage expectations, while psychosocial support, transitional counselling, and peer programs provided crucial coping mechanisms. Support should be offered proactively rather than sought by caregivers.	Finding suitable care facilities close to home was challenging, with good options often having long waitlists. Government services for accessing LTC were confusing, professional psychological support was limited, and staff had little time for private discussions, though monthly meetings were suggested as beneficial.
**6**	Chu 2020	Taiwan	57	Cross-sectional	Nursing home to ED Nursing home to hospital	NR	Provision of palliative care.	NR
**7**	Cottrell 2020	Canada	9	Qualitative	NR to institutionalization	NR	Supportive communities, shared caregiver experiences, gradual transitions, coordinated follow-ups, consistency in providers, familiarization with the LTC environment, proactive pretransition planning, compassionate health care providers, and joint decision making with the person living with dementia.	Slow transitions, system constraints prioritizing institutional needs, difficulty navigating the health care system, lack of provider knowledge, and insufficient information on the process.
**8**	Cronfalk 2018	Sweden	10	Qualitative	NR to residential aged care facility	People living with dementia: Adjustment to RACF varied, with many reporting poor acceptance after several months, compounded by feelings of community separation and loss of activities, physical health, and independence. Some experienced relief and security, while others felt trapped or resigned to the lack of alternatives. Greater acceptance was observed when individuals had a say in the admission process. Caregivers: observed distress and confusion in individuals with dementia within unfamiliar environments but felt relief from the safety and care provided. Despite this, they often found the process difficult and distressing, with relationship conflicts arising. Involving the person with dementia in relocation decisions and helping them understand the necessity of the move improved acceptance for both the individual and their family.	Support from family and staff, development of new relationships with other residents, provision of meaningful activities, and opportunities for autonomy were all critical in easing the transition.	NR
**9**	Damien 2020	Belgium	91	Case–control	Care home to acute hospital unit Own home to acute hospital unit Own home to hospital to care home All through ED Most cases required an ambulance	Families had to advocate assertively for individuals with dementia, facing long ED waits, frustration, and concerns about medication management. They were deeply involved in care, often staying with their loved ones, but advocacy efforts were emotionally taxing. Anxiety stemmed from ensuring dignity and dealing with inappropriate staff interventions, while families felt powerless and stressed by escalating situations.	Family advocacy, assertiveness, and presence were crucial in creating care plans, ensuring medication adherence, and discharge planning. Being listened to, effective communication and information sharing, and understanding how to approach a person with dementia contributed positively. Family presence had a therapeutic effect.	Poor communication and family members not being listened to created stress. Staff were often unfamiliar with dementia symptoms, including communication difficulties, and the hospital environment was not suitable for people living with dementia. Carers were underutilized, and there were concerns about undignified events, inadequate care, and systemic issues like fragmented care and inconsistent practices due to work pressures and time constraints.
**10**	Davison 2019	Australia	38	Qualitative	Emergency transition to hospital admission, transitions within primary care	NR	Higher continuity of care with the same GP led to fewer inappropriate prescriptions. Effective communication between health care providers and integrated care models, including electronic health records, improved care.	Low continuity of care, complex medication regimens with polypharmacy and inappropriate prescribing, system fragmentation, and resource limitations, such as limited GP time and availability, hindered effective care.
**11**	Delgado 2022	England	9,324	Cohort	Admission into GP, secondary care hospitals or ED	NR	Access to quality primary care, funding for mental health care, staff with dementia care experience, and improved geographic access to services facilitated transitions.	Deprivation, rurality, and socioeconomic factors led to higher admission rates in areas with limited services. Non-standardized referral criteria and inconsistent care practices between GP practices impacted the transition process.
**12**	deVries 2019	New Zealand	26	Qualitative	Home or hospital to nursing home	Families experienced difficult emotions during transitions, marked by feelings of failure and sadness. Over time, they reached a level of acceptance, believing they had done everything possible to support their loved one.	Professional 24-hr care, which is unsustainable at home, provided emotional relief knowing loved ones received appropriate care. Existing support networks, though sometimes insufficient, including home services, family members, and health professionals, assisted in the transition process.	Emotional challenges such as guilt, sadness, anger, shame, and fear accompanied the decision to move a loved one into a care home. A lack of information and support, along with stigma and social judgment, added to the stress. Caregivers felt inadequate health care guidance and had to independently seek out necessary information and support.
**13**	Eyles 2021	England	986,242	Cohort	From hospital to residential aged care	Emotional adjustment during transitions involved sadness, guilt, relief, and a sense of loss. Families found the search for care overwhelming and time-consuming, with hospital information delivery adding to the complexity. Despite these challenges, the placement experience, particularly with a dedicated dementia unit, was positively reflected upon due to good handover processes and quality care.	Professional support from social workers and health care providers, access to information about aged care options, emotional support networks for caregivers, and structured transition plans involving all stakeholders helped ease the transition process.	Lack of adequate or overwhelming information, emotional stress and guilt experienced by caregivers, financial constraints, poor coordination between care services (home care, hospital, residential aged care), and resistance from dementia patients to move to a new care setting were significant obstacles.
**14**	Fekonja 2021	Slovenia	19	Qualitative	Primary care to secondary or tertiary care: moving from home care to hospitals or specialized dementia care units Home care to residential aged care: transitioning from home or community-based care to nursing homes or residential aged care facilities Emergency department transitions: movement to emergency departments in cases of acute health episodes Respite care transitions: temporary transition to respite care facilities for short-term relief for primary caregivers	NR	Effective communication, support services, planning and coordination, training and education, and emotional support were key to facilitating transitions.	Lack of information, emotional resistance, financial constraints, inadequate support, and health system challenges hindered the process.
**15**	Fitzpatrick 2019	Australia	10	Qualitative	Home, hospital or community care to RACF	NR	Professional support, pretransition planning, emotional support, and clear communication between health care providers, family members, and the patient were essential to a successful transition.	Emotional resistance from family, financial constraints, lack of information about what to expect during the process, and inadequate or limited support services, such as respite, created significant challenges.
**16**	Freedman 2022	Canada	95	Quasi-experimental	Primary care to hospital, ED visits, and hospital readmission	Consistent care fostered stable relationships with health care providers, offering emotional support and reliable medical advice to better manage overall health.	High primary care continuity, integrated care systems, and active stakeholder involvement were crucial in ensuring effective care transitions.	Low primary care continuity, difficulties in accessing timely appointments, health system fragmentation leading to care gaps, and socioeconomic factors created barriers, with lower-income backgrounds facing greater challenges in accessing continuous, high-quality care, resulting in higher hospitalization rates.
**17**	Gilmore-Bykovskyi 2021	United States	298	Cohort	Home to residential aged care Changes in caregiver role: Transitioning from being a primary caregiver at home to a more supportive and advocacy role in the care home	Caregivers experienced an emotional burden, including stress, burnout, guilt, sadness, and loneliness. They faced a sense of loss and the need for adjustment but remained actively involved by advocating for and communicating with care home staff.	Professional support, information and resources about the transition process to support informed decisions, emotional support networks for family, friends, and support groups providing emotional relief and practical advice, and pretransition planning helped ease the process.	Emotional resistance, lack of information, financial constraints, inadequate support services, and negative perceptions of care homes created significant challenges.
**18**	Godard‐Sebillotte 2021	Canada	22,060	Cohort	Home to nursing home and interim housing to permanent nursing home	Families faced uncertainty and anxiety during transitions but found relief once individuals settled. Continuity in routines supported well-being, while concerns about death were often avoided.	Supportive nursing home environments, professional care, and the maintenance of routine were key to easing transitions.	Emotional resistance, multiple moves, and a lack of activities for residents created significant obstacles.
**19**	Gresham 2018	Australia	180	Quasi-experimental	Home to respite care and temporary respite to potential permanent placement	Caregiver training and respite care provided relief, reduced distress, and offered practical and psychological support, allowing caregivers to learn and share experiences. Individuals with dementia benefited from sensory and cognitive activities, improving engagement and supporting a structured routine.	A comprehensive training program offering psychological support, education, and practical home care skills, along with a supportive residential environment that provides safety and structure for both carers and people living with dementia, and professional multiskilled staff ensured comprehensive care and support.	Emotional resistance due to fear of the unknown, financial constraints, and logistical challenges, particularly when care is temporary, hindered the transition process.
**20**	Groenvynck 2022	Netherlands	24	Qualitative	Home to nursing home care or community to institutional care	Caregivers experienced relief and increased emotional loneliness from physical separation from their spouse. Social loneliness lessened as they regained some social activities.	Support from health care professionals, social support networks (strong family supports, friends and community groups) helps caregivers cope with the transitions, preparation and planning.	Emotional resistance from both carers and people living with dementia, financial constraints, lack of information and support to ease the process, cultural and social factors (cultural attitudes to nursing home care and social expectations).
**21**	Gustafsson 2018	Sweden	429	Randomized-control trial	Home to long-term care and temporary care to permanent placement	NR	Pharmacist reviews reduced PIMs, and collaboration among health care teams improved transitions. Regular medication monitoring during hospital stays and transitions enhances care.	High PIM prevalence increased risks, while undetected cognitive impairment and multiple chronic conditions complicated transitions and medication management.
**22**	Hähnel 2023	Germany	191	Randomized-control trial	Primary to secondary/tertiary care, home to long-term care and home to hospice care	Caregivers experienced initial relief and improved mental health after institutionalization, with reductions in depression and anxiety over time. The Tele.TAnDem intervention significantly improved caregivers’ quality of life and health after transitioning to a nursing home.	CBT improved mental health and quality of life for caregivers. Resource activation, focusing on enhancing problem-solving, stress management, and support service use, helped caregivers cope better.	NR
**23**	Hanson 2019	United States	62	Randomized-control trial	Hospital to home/assisted living/nursing home or emergency department visits	The intervention helped address emotional strain and decision making, particularly regarding prognosis, care goals, and treatment options. It also improved comfort for patients and provided better support for families through structured palliative care consultations and follow-up.	Specialty palliative care consultations provided comprehensive support, including symptom management and decision-making assistance. Postacute transitional care from palliative care nurse practitioners ensured continuity of care after hospital discharge. Advanced care planning through Medical Orders for Scope of Treatment forms clarified treatment preferences and goals.	Caregivers experienced emotional overwhelm due to the caregiving burden, making participation difficult. Short hospital stays limited the full implementation of palliative care interventions. Complex decision making about life-sustaining treatments and hospital readmissions required significant family support and guidance.
**24**	Hanssen 2019	Norway	45	Qualitative	Home to professional home care or home to nursing home	Family caregivers often sought professional help when they could no longer manage care responsibilities. The worsening condition of the person with dementia made home care unsafe or unmanageable. Additionally, concerns about negative reactions from other family members and the wider community contributed to delaying institutionalization.	Professional health care offers, including home and institutional care, provided vital support for caregivers struggling alone. Culturally tailored information and support helped families accept professional care as a viable option.	Cultural expectations in collectivistic societies strongly emphasized that family members should provide care themselves. This led to feelings of stigma, shame, and fear of community judgment for not fulfilling familial duties. The moral obligation to care for parents made the decision to seek professional help especially challenging.
**25**	Hanssen 2022	Norway	102	Qualitative	Home to nursing home	Caregivers of people living with dementia often experience emotional strain, including shame and stigma, particularly in cultures that emphasize familial duty. Many feel predecision regret, torn between personal needs and societal expectations, and struggle with guilt and fear of judgment when deciding to institutionalize their dependents.	Professional guidance from health care providers was crucial in supporting caregivers through the decision-making process. The availability of formal and informal services helped caregivers understand their dependents’ needs and navigate transitions. Culturally sensitive support tailored to caregivers’ expectations eased the process.	In collectivistic cultures, strong societal and familial expectations to care for parents at home made seeking professional care difficult. Feelings of guilt, shame, and the stigma associated with placing a family member in a nursing home contributed to caregivers’ reluctance to seek professional help.
**26**	Harkin 2020	Australia	126	Qualitative	Home to respite cottage or home to traditional RACF respite	Carers reported high satisfaction with the cottage model of respite care, appreciating its homely and intimate environment compared to traditional RACFs. Many found it provided much-needed emotional relief and rest, enabling them to continue caregiving at home for longer. The familiar, non-institutional setting also positively impacted care recipients’ well-being, easing the transition for both carers and care recipients.	This model helped carers maintain their caregiving roles longer and delayed the need for permanent residential care. Expanding the availability of cottage respite services could provide more families with access to this effective and preferred model of care.	Limited availability, awareness, access, and the cost of cottage care were significant barriers to utilizing this model.
**27**	Harrison 2023	United States	463,947	Cohort	Home to nursing home	NR	Professional support, structured plans, and community and social support provided through the home and community support program reduced the likelihood of nursing home use.	Financial concerns and a lack of information were significant barriers to accessing the program.
**28**	Holton 2023	Ireland	11	Qualitative	Home to nursing home care or community to institutional care	A mixture of relief and increased loneliness, emotional loneliness in particular, due to the physical separation from the spouse. Social loneliness may decrease as the caregiver regains some social life activities.	Support from health care professionals and social support networks, including strong family, friends, and community groups, helped caregivers manage transitions, preparation, and planning.	Emotional resistance from both carers and people living with dementia, financial constraints, lack of information and support, as well as cultural and social factors such as attitudes towards nursing home care and social expectations, created significant challenges.
**29**	Hui 2022	China	17	Qualitative	Home to long-term care and temporary care to permanent placement	Support from health care professionals for guidance and assistance, social support from family, friends, and community, along with preparation and planning, helped ease the transition. Cultural sensitivity, addressing cultural expectations and stigmas associated with long-term care placement, was also key.	Emotional resistance from both the carer and people living with dementia, financial constraints, and lack of information and support, including high costs and insufficient details, hindered the process. Cultural stigma, driven by negative societal attitudes and traditional beliefs about home caregiving, compounded caregiver stress and guilt.	High initial burden: caregivers who institutionalize their relatives start with high levels of burden, anxiety, and depression Complex feelings and emotions: guilt, grief and loss were prevalent during the transition
**30**	Jennings 2020	United States	3,995	Quasi-experimental	Primary to secondary/tertiary care, home to long-term care and home to hospice care	Structured support provided emotional relief to caregivers through continuous access to assistance and advice. Patients in the program were more likely to utilize hospice services, facilitating smoother transitions to end-of-life care.	Nurse practitioner involvement, comprehensive and regularly updated care plans, and support systems played a key role in ensuring effective transitions.	Medical comorbidities, caregiver burden (both physical and emotional), and health care system fragmentation made the transition process more complex.
**31**	Johannessen 2019	Norway	10	Qualitative	Home to supported living accommodation or a nursing home	Receiving a dementia diagnosis was often described as a dramatic and distressing event, evoking fear about the future. Over time, many individuals adapted through cognitive and emotion-focused coping, achieving a good quality of life. Similarly, while moving to residential care was initially met with reluctance and anxiety, it was later perceived positively by many, offering safety, social interaction, and an improved quality of life compared to living alone.	High-quality public support, including well-structured public health services, along with social support and activities, helped participants feel positive about the transition. Professional guidance, with continuous support, regular check-ups, and personalized care plans, further supported the process.	Emotional resistance, stigma, social perceptions, and the loss of independence were significant obstacles in the transition.
**32**	Kable 2019	Australia	277	Quasi-experimental	Home to hospital or RACF to hospital	NR	Hospitalization provided an opportunity for medication review and adjustment, reducing the number of medications and PIM. Health care professionals, including pharmacists, were involved in identifying and addressing medication-related issues during hospital stays.	Inadequate detection of cognitive status during admission led to potential medication-related problems. The high prevalence of polypharmacy and PIMs at the time of admission complicated patient care.
**33**	Ke-Hau 2020	Taiwan	80	Cohort	Home to hospital, long-term care or respite	Exercise is associated with lower rates of unexpected hospitalization, supporting better health management and reducing reliance on emergency care.	Regular exercise programs significantly improve physical health, reducing the need for hospital admissions. Continuous physical fitness assessments helped in the early identification and management of health issues. Support from health care providers in guiding exercise programs led to better health outcomes.	Cognitive decline, particularly in moderate to severe dementia, made maintaining physical fitness and managing health independently challenging, leading to more frequent hospitalizations. Lack of consistent exercise and inadequate health care system resources for implementing exercise programs and fitness assessments hindered effective health management.
**34**	Khemai 2022	Netherlands	32	Qualitative	Home to nursing home, transitions within nursing homes, hospital to nursing homes	Informal caregivers faced significant emotional strain during care transitions, particularly when moving loved ones to nursing homes. Over time, many adapted and played essential roles in providing continuous, personalized care. They were also actively involved in sharing information about patient preferences and needs and in making decisions during care transitions.	Proactive health care professionals who engage with caregivers, provide timely information, and involve them in decision making were crucial. Effective communication channels, including phone calls, emails, and face-to-face meetings, ensured caregivers stayed informed. A consistent first contact person, such as a case manager or primary nurse, guided caregivers through the transition, supported by structured support systems like multidisciplinary meetings within nursing homes.	Lack of communication and untimely information exchange led to gaps in care continuity. Emotional resistance, guilt, and stress made it difficult for caregivers to accept the need for transition. Inconsistent care practices and high staff turnover complicated care, leading to confusion and suboptimal patient outcomes.
**35**	Kupeli 2019	England	14	Qualitative	Home to care home or hospital to care home	Emotional resistance, including difficulty accepting institutional care and feelings of guilt and failure among family carers, was prevalent.	Professional support helped guide families through the transition process, providing necessary assistance. Advance care planning, including Do Not Attempt Resuscitation orders, clarified care preferences and reduced uncertainty. Access to resources such as the Gold Standards Framework for end-of-life care, along with staff training in dementia care, facilitated smoother transitions.	Financial constraints, lack of information, and inconsistent care quality also posed significant challenges.
**36**	Larsen 2020	Norway	12	Qualitative	Home to nursing home	The decision-making process was complex, requiring caregivers to balance the needs of the person with dementia with their own. Caregivers navigated various roles, such as self-condemning determiner or dominant, each presenting unique emotional and practical challenges.	Effective involvement and support from health care professionals guided family caregivers through the decision-making process. When the health deterioration of the person with dementia made nursing home admission necessary, it helped legitimize the decision in the eyes of caregivers and others involved.	Emotional resistance made it difficult for both the person with dementia and their family members to accept the need for nursing home care. Many caregivers felt unprepared for the decision-making process and the transition itself. Conflicts with health care providers over the best course of action, along with feelings of perceived exploitation due to excessive reliance on caregivers, further complicated the process.
**37**	Leggett 2018	United States	652	Cross-sectional	Home to ED or home to hospital	Caregivers of persons with dementia experienced significant emotional strain, particularly after falls, which heightened stress, anxiety, and feelings of being overwhelmed. Falls increased caregiver vigilance and fear of recurrence. Many caregivers faced role overload, exacerbating the emotional challenges during transitions.	Support from family and friends helped reduce stress during transitions. Caregiving gains, such as increased confidence and a stronger bond with the care recipient, helped mitigate emotional difficulties.	Many caregivers were unprepared for sudden events, like falls, which increased stress. A lack of information and resources made managing care transitions more challenging, both emotionally and logistically. Family disagreements over care decisions also heightened emotional difficulty for the primary caregiver.
**38**	Lei 2020	United States	105,528	Cohort	Home to hospital Nursing home/SNF to hospital Assisted living facility to hospital	NR	Support from health care providers and access to medical information, along with continuity of care, positively impacted hospitalization. The physician’s accumulated knowledge of the patient’s condition, coupled with a trusting and responsible provider-patient relationship, contributed to better care.	Continuity of care did not improve ambulatory care-sensitive ACSC, and current treatment guidelines for ACSC hospitalizations, which emphasize patient self-management, were problematic for patients with dementia.
**39**	Liu 2021	United States	250	Mixed-methods	Primary to specialized care	Caregivers often felt overwhelmed by the unclear implications of a dementia diagnosis, expressing concerns about daily care, social and financial support, and the need for a safe space to share their stress. Memory clinics had a positive impact, offering educational and social support that reduced isolation and provided personalized attention through dementia navigators. Caregivers also highlighted the importance of self-care, with the memory clinic playing a vital role in supporting their well-being to ensure better care for their loved ones.	The dementia navigator provided continuous support and acted as the primary contact for caregivers. A multidisciplinary team, including a geriatric physician, nurse, nurse practitioner, and social worker, ensured comprehensive care. Educational and social support, along with detailed information about dementia and assistance navigating the medical system, further supported caregivers.	Caregivers initially felt overwhelmed by the diagnosis and its implications, making it difficult to navigate the system and access necessary support. Despite the support from the memory clinic, some caregivers still struggled with accessing resources and managing daily caregiving challenges.
**40**	Manis 2021	Canada	977	Cohort	ALF to nursing home	NR	The rate of transition to a nursing home was significantly lower among older adults who resided in an assisted living facility that offered a dementia care program.	NR
**41**	Marsh 2023	Australia	38	Qualitative	Home to RACF or a community day center to RACF	Increased care needs caused significant emotional strain on family caregivers. Relocation stress was alleviated by fostering strong relationships between caregivers at home and those in RACFs.	Creative expression facilitated social and emotional outcomes by allowing individuals to share their stories and express their identities. Professional support from both health care and creative professionals enhanced care. The visibility and communication fostered by films created through the project improved understanding between care staff and new residents, aiding smoother transitions.	Emotional resistance from both people living with dementia and their families in accepting the need for care posed a challenge. Privacy concerns arose, with some participants worried about public exposure. Resource constraints, including limited time, staff, and technological limitations in care settings, hindered the full utilization of films for relational care.
**42**	Mogan 2022	United Kingdom	29	Qualitative	Home to hospital via EMS or home care	Four key themes emerged in dementia care transitions: poor continuity of care, lack of expertise, limited advance care planning, and loss of autonomy. Caregivers valued ongoing relationships with professionals, which preserved the dignity of the person with dementia, but frequent changes in care workers were unsettling, particularly for personal care. A lack of consistent monitoring caused confusion and stress in accessing services. Poor communication between health and social care services often led to inappropriate interventions, including distressing situations like unnecessary resuscitation attempts.	Caregivers reported positive experiences when there was good relational continuity and support from knowledgeable professionals, such as Memory Clinic nurses and Admiral Nurses. Their expertise and emotional support enabled some caregivers to keep the person with dementia at home until the end.	Lack of expertise in dementia and end-of-life care hindered effective transitions. Limited advanced care planning left caregivers unaware of the process, creating uncertainty and stress. Caregivers also experienced a loss of autonomy, feeling controlled by health and social care services, which hindered open communication and collaborative decision making.
**43**	Nguyen 2022	United States	303	Mixed-methods	Home to home-based primary care	Home-based primary care was praised by family caregivers for its coordinated, continuous, and convenient care, aligning with their priorities and often exceeding expectations by effectively managing care at home and reducing clinic visits. The HBPC team provided valuable emotional and informational support, helping caregivers navigate the challenges of their role. However, caregivers faced difficulties accessing personal care assistance for tasks like bathing, toileting, and transferring patients, as well as covering the costs of medical supplies and equipment.	Family caregivers reported that home-based primary care provided coordinated, continuous, and convenient care, aligning with their priorities and goals. Emotional and informational support from the HBPC team helped caregivers cope with caregiving demands. The involvement of interdisciplinary teams, including geriatric and palliative care professionals, and access to home-based laboratory, radiology, and skilled nursing services, facilitated the transition to HBPC. After-hours triage and support from palliative care nurses and on-call physicians provided urgent medical advice when needed.	Despite positive experiences with medical care, caregivers faced challenges in obtaining assistance with personal care tasks, such as bathing, toileting, and transferring patients, as well as with paying for medical supplies and equipment.
**44**	Parker 2021	Australia	60	Cohort	From hospital to a nursing home	Transitions for older adults with dementia reveal significant gaps in addressing their physical, social, and behavioral needs. Poor documentation of dementia symptoms and cognitive impairments hampers person-centered care during discharge. The study highlights the vulnerability of frail older adults, particularly those with limited or no verbal communication, who often depend on others to advocate for their needs.	High-quality discharge documentation, effective interprofessional communication, and eHealth system integration facilitated smoother transitions and better coordination of care.	Poor documentation quality and limited education and information hindered effective care transitions. Discharge documentation often failed to anticipate future care needs or the capacity of nursing homes to address them. A lack of standardization in discharge processes across health districts or states contributed to fragmented care and communication gaps.
**45**	Pocock 2018	United Kingdom	18,404	Cross-sectional	Transport to ED via an EMS ambulance	NR	High-quality discharge documentation, effective interprofessional communication, and eHealth system integration facilitated smoother transitions and better coordination of care.	Poor documentation quality and limited education and information hindered effective care transitions. Discharge documentation often failed to anticipate future care needs or the capacity of nursing homes to address them. A lack of standardization in discharge processes across health districts or states contributed to fragmented care and communication gaps.
**46**	Pond 2019	Australia	73	Cross-sectional	Hospital to home	NR	Discharge documents that referred carers to specific support or education groups were beneficial. A higher compliance score for items provided to GPs at discharge was linked to decreased odds of readmission, though not statistically significant.	Discharge summaries were often incomplete, with only half documenting routine assessments for confusion, falls, and pressure injury risks. One-third of discharge summaries lacked documentation of a week’s supply of medications. Medication dose-decision aids were offered in only half of the cases, and there was a lack of contact information for patient support groups or advance care planning. Pending test results, ongoing clinical issues, and missing information in medical summaries hindered continuity. Carer education on managing exacerbations and signs of complications was often inadequate, and home medication reviews were rarely recommended. Additionally, there were variable onward referrals and appointments.
**47**	Prusaczyk 2019	United States	126	Cross-sectional	Home/facility to hospital	NR	Caregivers expressed a desire for more education and information from hospital providers to better advocate for patients in aftercare. Educating caregivers of persons living with dementia has been shown to ease caregiver burden.	Lack of sufficient education and information from hospital providers made it difficult for caregivers to advocate effectively for patients’ aftercare needs.
**48**	Prusaczyk 2020	United States	126	Mixed-methods	NR to hospital	NR	Clear delineation of roles within a multidisciplinary team, where social workers and case managers handle discharge planning and psychosocial support, while nurses and physicians focus on education, medication safety, and discharge communication. Structured processes, such as triaging responsibilities, and alignment with established frameworks further support effective care transitions.	Poor comprehension of discharge instructions and behavioral issues, which complicate care coordination. Provider limitations, inconsistent documentation, and gaps in tailored interventions for dementia patients exacerbate these issues. Additionally, variation in practices across care units and a lack of dementia-inclusive transitional care models hinder the consistency and effectiveness of care transitions for this population.
**49**	Radcliffe 2023	United States	31	Qualitative	Home to LTC	Overwhelming uncertainty about care needs, unsure when to plan for additional support. Many express a desire to understand “what stage we are at” in the progression of dementia but lack a clear plan to keep their loved ones at home, leaving them uncertain about future care. This uncertainty is compounded by feelings of isolation.	Carers sought guidance from health care professionals regarding institutional placement or support to stay at home, particularly as the disease progressed. Early and specific guidance related to transitions between home and LTC was valuable. Counselling on prognosis and expected disease course helped carers plan early, while later guidance helped identify care locations and resources. Prognostic information about disease stage and life expectancy helped carers make informed decisions about care settings. Proactive and early guidance from HCPs and advice from primary care providers facilitated home care and aided carers in preparing for potential transitions to LTC.	Carers were unsure about when to plan for care transitions, particularly for LTC placement, and struggled with deciding when the care situation was dire enough for institutionalization. Disparaging comments from physicians often made it more difficult to navigate tough decisions regarding care settings.
**50**	Rapp 2018	France	686	Cohort	Home to short/long-term care	Permanent stays in care facilities can improve certain aspects of patients’ well-being by reducing the risk of agitation and irritability.	A permanent stay in LTC becomes more effective when patients experience intense behavioral issues, increased functional dependence, and greater cognitive decline.	NR
**51**	Rognstad 2020	Norway	11	Qualitative	NR	Difficulties finding suitable nursing homes leading to multiple relocations. Decisions to move people living with dementia, often against their will, are emotionally fraught for caregivers, who struggle to communicate the need for more care. Health care authorities typically make final decisions, adding to the complexity. People living with dementia may resist transitions, viewing others as sicker, expressing a desire to return home, or attempting to leave, which heightens caregivers’ feelings of guilt and ambivalence.	A hospital with a day-care center for FTD patients provided trained staff to manage behavior, highly individualized activities, transport home, and the option for people living with dementia to stay overnight in a “relief department.” Carers benefited from monthly meetings with a nurse and psychologist to prevent and manage issues, while identifying and supporting the needs of people living with dementia.	Finding institutions capable of navigating behavioral challenges and offering person-centered activities was difficult. Tensions between carers and people living with dementia over entering LTC could escalate, leading to immediate professional intervention and complaints from people living with dementia. There was a lack of information about nursing homes and day-care centers, and staff often lacked the experience to manage aggressive behavior. Low staffing levels and insensitive health care providers compounded these issues, with limited explanations about moves between care facilities. Additionally, people living with dementia experienced isolation and inadequate participation in activities due to the focus on physically ill patients, and FTD patients required different resources and care approaches not available in many nursing homes.
**52**	Rubinsztein 2020	United Kingdom	171	Mixed-methods	Community to hospital	NR	Advice and support for carers, pharmacological and non-psychological interventions for BPSD, referrals for increased social support or respite, and supportive counselling for both carers and patients helped improve care. Recommendations for changes in physical health medication, fall prevention, delirium assessment, and management also contributed to better care. Educational programs for care homes and benefits advice to carers further supported the care process.	NR
**53**	Ryvicker 2022	United States	56,652	Cohort	NR to home health care	NR	A standard mechanism for transferring relevant medical information between providers ensured smoother care transitions and better communication.	Poor documentation, lack of integrated health care records, and inadequacies in transferring critical information from previous health care settings to the next team hindered effective care coordination.
**54**	Saragosa 2023	Canada	25	Qualitative	Hospital to home	Emotionally and practically challenging for caregivers, involving managing medications, home adaptations, and unanticipated declines in functioning. Cognitive deterioration during hospital stays often pressures families to bring loved ones home, despite feeling unprepared. Caregivers face uncertainty, guilt, and exhaustion, frequently advocating for support while feeling isolated. Over time, the caregiving burden and declining health may force acceptance of long-term care, with transitions marked by high demands, emotional strain, and a sense of losing control.	Having a supportive health care professional (“cheerleader”) knowledgeable about dementia helped build trust between staff and families at critical transition points. Home care was smoothly arranged from the hospital with minimal disruption, and services resumed upon discharge. Knowing whom to call for support provided reassurance during the transition.	Lack of clarity around the discharge plan, including criteria for discharge and what to expect at home, led to confusion. Caregivers faced uncertainty about their own capacity to care and anxiety about preparing meals in line with new diets. They had to advocate strongly for care while managing complex needs at home, including medication administration, feeding, and wound care. Posttransition, care needs increased drastically, requiring 24/7 supervision, and balancing these needs with other responsibilities, such as work, was overwhelming. Immediate care coordination postdischarge was essential, but caregivers often had to learn how to access and manage home care services on their own. Inadequate training in dementia care for home and community service providers, lack of engagement in advanced care planning, and the need to “learn as you go” made the process more challenging.
**55**	Sawan 2021	Australia	31	Qualitative	Hospital to home/LTC/Assisted living	Caregivers often feel overwhelmed by discharge processes, with inadequate counselling on medications and a need to independently seek information on medication-related harm. Many take on the responsibility of creating medication lists due to poor documentation, feeling overlooked by hospital staff who prioritize communication with long-term care facilities. The pressure to ensure timely medication administration adds to their stress and burden. Managing the emotional needs of people living with dementia and addressing symptom exacerbation from the unfamiliar hospital environment further intensifies caregiver fatigue and strain.	A tailored medication list, including information on medications impacting cognition, and staff communication with community pharmacists and primary care physicians helped improve coordination posttransition. Hospital pharmacies communicating directly with community pharmacists ensured medication changes were implemented correctly. Additional support during and postdischarge facilitated better engagement and planning for medication management.	Inadequate information about medication management at discharge, limited caregiver involvement in decisions, and difficulties ensuring medication supply postdischarge were significant challenges. Conversations during discharge were brief, focusing only on medication supply, without discussing risks, benefits, adverse effects, or treatment duration. The discharge summary was often too lengthy, dense, and filled with technical jargon. There was a lack of timely information exchange between the hospital, primary care physician, or long-term care facility, and discharge summaries were sometimes inconsistent or not updated with the hospital treatment plan. Inadequate medication documentation, difficulty organizing prescriptions during weekends, and caregivers being absent due to unplanned discharges further complicated the process.
**56**	Sharda 2020	United States	927	Cohort	Home to hospital Nursing home/SNF to hospital Assisted living facility to hospital	NR	IPCCs facilitate meaningful goals-of-care discussions, aligning treatment with patient and family preferences. They improve communication, increase hospice enrolment, and enhance end-of-life planning through DNAR orders. IPCCs focus on advanced dementia patients, addressing symptom management and holistic needs, with longer hospital stays providing time for comprehensive care planning.	Barriers include underutilization of palliative services, limited access due to systemic and financial constraints, and a focus on acute conditions during hospitalizations. Late introduction of palliative care and insufficient awareness among providers and families hinder early symptom management and consistent care approaches.
**57**	Smith 2022	Australia	18	Qualitative	Existing site to another new facility	Care transitions cause significant distress for people with dementia, including worries about changes in routine, difficulties establishing new routines, and disruptions to sleep patterns.	Increased access to enjoyable activities, a physical environment with direct access to nature, privacy, and comfort, and more staff providing one-on-one support helped improve care. Person-centered objects, supporting personhood, and effective wayfinding also contributed to a positive experience. Communication and technology enhanced care, while meaningful activities and continuity created stability. Fostering connections through community, volunteers, family, and staff helped in making the environment feel more like home.	NR
**58**	Smith 2023	Australia	15	Qualitative	RACF to new purpose built facility	Care transitions can be highly distressing for people living with dementia, with stress over packing personal belongings, fears of material loss, and concerns for their safety. These transitions often provoke emotional and physical reactions, such as crying, screaming, and resisting.	Personalized scarves and blankets provided comfort during physical transitions, offering a sense of familiarity and warmth.	Not having usual items (e.g., handbags) or familiar people (e.g., family) during the move caused distress and a sense of loss.
**59**	Statz 2022	United States	83	Mixed-methods	NR to RACF	Care transitions for people living with dementia evoke complex feelings of guilt for caregivers. Many question their decision, feeling guilt is heightened if people living with dementia resist the move or if they believe they could have tried harder to keep them at home. The timing of the move—whether too early or too late—also affects guilt levels. Caregivers feel less guilt if the transition is proactive or enables socialization for people living with dementia but more if they perceive it worsens dementia. External judgments from family and friends amplify guilt, as does poor caregiver health or work obligations limiting visits.	High-quality care homes.	Personal finances.
**60**	Toles 2022a	United States	19	Qualitative	SNF to home of people living with dementia SNF to assisted living SNF to home of carer	NR	Having someone who listens, offers advice, and affirms that caregivers are making the right decisions provided emotional support. Familiarity, flexibility for appointments, and written information also helped, along with a commitment to adhering to agreed plans.	NR
**61**	Toles 2022b	United States	13	Qualitative	SNF to home SNF to assisted living	Caregivers often face an unmet need for a clear “dementia care plan,” leaving them unprepared for dementia symptoms, medication management, and postdischarge issues like falls. Many feel uncertain, frantic, and exhausted, compounded by discomfort in asking for help.	NR	People living with dementia and caregivers may not be ready to fully engage in dementia care planning while in the SNF. Caregivers are often unprepared to manage dementia symptoms at home. SNF staff face challenges in connecting PWD and caregivers to community supports, and caregivers receive little support to address their own needs.
**62**	Tropea 2022	Australia	1,304	Mixed-methods	Nursing home to hospital	NR	Simulation-based training which enhances staff skills in dementia-specific palliative care through realistic scenarios and accessible formats.	Competing organizational priorities, staff workload, and limited engagement significantly undermine the impact of such interventions.
**63**	van der Heide 2021	Netherlands	1,110	Cohort	NR	Transitions during the dementia care trajectory—such as diagnosis, institutionalization, and the death of the person with dementia—have a profound impact on the health and socialization of cohabiting partners.	Health care professionals, particularly GPs, play a critical role in identifying and addressing caregiver health issues early. Support services like home care and dementia case management can alleviate caregiver burden, with institutionalization sometimes offering relief by reducing physical caregiving demands.	Health care professionals, particularly GPs, play a critical role in identifying and addressing caregiver health issues early. Support services like home care and dementia case management can alleviate caregiver burden, with institutionalization sometimes offering relief by reducing physical caregiving demands.
**64**	Wang 2022a	United States	357,641	Cohort	NR to hospital	The combination of telehealth postdischarge and enabling services is an important intervention to reduce preventable hospitalizations among patients with ADRD living in rural and micropolitan areas.	NR	Lower-rated SNFs
**65**	Wang 2022b	United States	2,011,351	Cohort	SNF to hospital	NR	The National Partnership to Improve Dementia Care in Nursing Homes facilitated a decline in antipsychotic medication use, promoting safer and more person-centered dementia care. This effort correlated with a modest reduction in 30-day hospital readmissions for SNF residents, particularly in high-quality facilities.	Limited capacity of low-quality nursing homes to implement non-pharmacological interventions due to resource constraints. While high-quality facilities benefited significantly, lower-quality facilities lacked the staff training and infrastructure needed to address behavioral challenges effectively, leading to uneven outcomes.
**66**	Wright 2023	New Zealand	18	Qualitative	NR to RACF	Health care professional recommendations for care transitions were seen as positive, alleviating caregiver guilt by shifting responsibility for the decision. Despite this, caregivers often resisted transitions, even when their own well-being was compromised, viewing residential care as a last resort. The decision-making process was difficult, with initial resistance followed by reflection and eventual acceptance that it was the right choice, though doubts often lingered.	Trust in professional judgment, receiving a doctor’s recommendation to act promptly, family reassurance, and re-reading personal diaries to remember the necessity of the transition helped ease the decision-making process.	When the doctor’s recommendation came during a crisis, the decision felt rushed. Attempts at respite care were counter-intuitive, often leaving caregivers exhausted. It was not until a crisis, such as a hospital admission, that RACF options were seriously considered.
**67**	Zmora 2021	United States	85	Mixed-methods	NR to RACF	Caregivers varied in their confidence when interacting with staff during care transitions. Some felt comfortable raising concerns and making suggestions, while others struggled to be assertive. Many were uncertain whether their expectations were realistic or if staff behaviors were typical.	Consistent and timely communication, strong relationships with trusted staff, and a detailed understanding of personal history, family, personality, and preferences enhanced care. Care conferences, collaboration to brainstorm solutions, and staff warmth and compassion supported both residents and caregivers. Positive staff attitudes, respect, dignity, and responsiveness to caregiver suggestions contributed to a more comfortable care environment. Infrastructure elements such as the physical environment, available care levels, and activities provided also played a key role in successful transitions.	Inconsistent or frantic phone calls with incomplete information caused stress. Uncertainty about which staff member to contact led to frustration, while a lack of communication between staff and external providers created confusion. Concerns over safety, falls, wandering, abuse, neglect, ADLs, and medication management affected perceptions of care. Low staff competency and insufficient training in dementia care, high staff turnover, and understaffing further complicated transitions. Financial limitations, such as long-term care insurance or Medicaid, often restricted available community and care options.

*Note*. ACSC = ambulatory care sensitive conditions; ADL = activities of daily living; BPSD = behavioral and psychological symptoms of dementia; CBT = cognitive behavior therapy; DLB = dementia with Lewy bodies; FTD = frontotemporal dementia; ED = emergency department; IPCCs = Inpatient palliative care consultations; LTC = long-term care; NR = not reported; PIM = potentially inappropriate medication; RACF = residential aged care facility; SNF = skilled nursing facility.

**Table 2.** Overview of themes and subthemes from the thematic synthesis.

Theme	Subthemes	Brief explanation
**Theme 1. Systemic and structural influences on transitions**	1.1 Health system complexity and fragmentation 1.2 Information gaps and system navigation challenges 1.3 Medication management across transitions 1.4 Financial and policy constraints 1.5 Structural and system-level facilitators and innovations supporting transitions	Systemic factors (policy, governance, funding) and structural factors (organization, coordination of care) both shaped transition experiences. Transitions were shaped by the organization and functioning of health care systems. Barriers included fragmented services, inequitable access, and poor information sharing. Facilitators included proactive planning, integrated care models, and supportive policy frameworks.
**Theme 2. The role of the health and social care workforce in transitions**	2.1 Gaps in dementia-specific knowledge and training 2.2 Empathy, communication, and staff attitudes 2.3 Staffing pressures and continuity challenges	Workforce skills, empathy, and communication strongly influenced transition quality. Limited dementia-specific training, staff shortages, and high turnover hindered care, while consistent, compassionate staff improved experiences.
**Theme 3. Caregiving, emotions, and decision making in transitions**	3.1 Knowledge and support networks 3.2 Caregiver advocacy and role recognition 3.3 Emotions about timing and decision making 3.4 Navigating facility selection and residential transitions 3.5 Emotional adjustment, conflict, and identity	Caregivers faced emotional, informational, and decision-making challenges throughout transitions. Support networks, early guidance, and involvement in planning reduced stress, while poorly timed or unsupported transitions intensified distress and guilt.
**Theme 4. Cultural, social, and situational influences on transition pathways**	4.1 Cultural beliefs, stigma, and moral expectations	Cultural values, social norms, and moral obligations shaped attitudes toward care transitions. Stigma, limited culturally tailored services, and moral expectations around family caregiving affected decision making and access to support.

Critical appraisal revealed both strengths and limitations across study types (supplementary Appendix C). The qualitative studies demonstrated strong methodological congruence between the stated philosophical perspective, research methodology, and data collection (*n *= 31); however, significant gaps were identified, with most studies failing to state the researchers culturally or theoretically perspective (*n *= 26) or address their influence on the research process (*n *= 29). Cohort studies performed well in reliably identifying exposures and measuring outcomes (*n *= 17), yet the majority struggled to adequately identify or manage confounding factors (*n *= 14). Quasi-experimental studies provided clarity in cause-and-effect relationships and reliable outcome measurement but frequently lacked control groups (*n *= 2). RCTs exhibited methodological weaknesses, particularly in allocation concealment (n = 4), blinding (*n *= 6), and outcome measurement reliability (*n *= 7), although statistical analyses were generally robust. Cross-sectional studies were strong in defining inclusion criteria and describing study settings (*n *= 7) but often failed to address confounding factors (*n *= 5). The single case–control study demonstrated overall methodological soundness but similarly failed to identify or manage confounding variables (*n *= 1). These findings underscore the importance of addressing methodological limitations, particularly confounding in quantitative designs and reflexivity in qualitative research, to strengthen the overall evidence base.

A summary of the number of individual studies that reported on specific barriers and facilitators can be found in supplementary Appendix D.

Thematic synthesis of the 67 included studies is presented later. The analysis is organized into four themes that illustrate how barriers, facilitators, and lived experiences shape care transitions for people living with dementia and their informal caregivers: (1) systemic and structural influences on transitions; (2) the role of the health and social care workforce in transitions; (3) caregiving, emotions, and decision making in transitions; and (4) cultural, social, and situational influences on transition pathways. Together, these themes reflect the interplay between the broader systemic environment, encompassing governance, policy, and funding, and the structural organization of care delivery and coordination, alongside the interpersonal and contextual factors that shape transition experiences (Bronfenbrenner & Morris, 1998; Donabedian, 2005). See Table 2 for an overview of themes and subthemes from the thematic synthesis.

## Theme 1: systemic and structural influences on transitions

### How policy, governance, and service-design factors hinder or enable care transitions

Transitions were influenced by both systemic and structural factors that interacted to shape the experiences of people living with dementia and their caregivers. Systemic factors refer to the broader policy, governance, and funding environments that determine how dementia care transitions are supported, while structural factors relate to the coordination and delivery of health and social care services across settings. Across studies, both levels of influence were evident; systemic issues such as policy fragmentation and resource limitations often compounded structural barriers like poor coordination, inadequate communication, and service discontinuity. Successful transitions were supported by coordinated efforts such as timely follow-up care, consistent referrals, efficient information exchange, personalized care planning, and interprofessional collaboration (Ashbourne et al., 2021; Delgado et al., 2022; Fekonja et al., 2021; Fitzpatrick & Grace, 2019; Freedman et al., 2022; Gilmore-Bykovskyi et al., 2021; Groenvynck et al., 2022; Gustafsson et al., 2018; Hanssen & Tran, 2019; Harrison et al., 2023; Johannessen et al., 2019; Khemai et al., 2022; Pond et al., 2019; Rognstad et al., 2020; Rubinsztein et al., 2020; Sawan et al., 2021; Smith et al., 2021; Toles et al., 2022a). However, many studies described systemic shortcomings that hindered these structural processes and negatively impacted both people with dementia and their caregivers.

#### Health system complexity and fragmentation

Structural fragmentation within health care systems, exacerbated by systemic inconsistencies in policy and funding, was a major barrier to continuity of care. Health care systems were often described as fragmented, particularly in contexts where services spanned multiple providers or where private and public care were poorly integrated. Caregivers reported difficulties navigating decentralized systems, often encountering conflicting information or disjointed care when transitioning between hospital, home, or long-term care settings (Groenvynck et al., 2022; Hanson et al., 2019; Smith et al., 2021). These barriers led to delayed transitions, inadequate discharge planning, and breakdowns in continuity of care. Contributing to this fragmentation was the lack of integration between electronic health records (EHRs) across health and social care providers, which hindered effective information exchange during transitions (Delgado et al., 2022; Parker et al., 2021; Pocock et al., 2018; Ryvicker et al., 2022). The inconsistency in how dementia diagnoses were recorded in EHRs further complicated care continuity, as health care professionals were often unaware of a person’s diagnosis or care needs (Pocock et al., 2018).

#### Information gaps and system navigation challenges

At both the systemic and structural levels, gaps in information flow and support systems made care transitions confusing and burdensome for families. Despite the implementation of support initiatives intended to simplify care transitions, such as Australia’s informational gateway services, caregivers reported that these were helpful in intent but insufficient in practice, often confusing, bureaucratic, and difficult to navigate during already stressful periods, with limited individualized or timely support (Brooks et al., 2022a; Fitzpatrick & Grace, 2019). Despite the implementation of support initiatives intended to simplify care transitions, such as Australia’s informational gateway services, caregivers frequently reported that these systems were overly complex and difficult to navigate in practice. Many caregivers felt that existing health care guidance was insufficient and experienced a lack of clear, coordinated information, often needing to “connect the dots” themselves to understand what services were available or how to access them (Fekonja et al., 2021; Saragosa et al., 2023). Specific information gaps were noted around options for long-term care, day-care centers, palliative and hospice services, and in-home support (Armstrong et al., 2020; Fitzpatrick & Grace, 2019; Kupeli et al., 2019; Larsen et al., 2020; Radcliffe et al., 2023; Rognstad et al., 2020).

System navigation was further complicated by burdensome paperwork, often required during times of high emotional stress (Brooks et al., 2022b; de Vries et al., 2019; Sawan et al., 2021). This administrative load, combined with inconsistent service coordination, left many caregivers feeling overwhelmed and unsupported. Caregivers often experienced conflicts between systemic pressures (e.g., lack of funding for services) and the care needs of their loved ones, with some caregivers expressing concerns that local health care services excessively relied on them to continue caring, leading to feelings of exploitation (Ashbourne et al., 2021; Larsen et al., 2020). Furthermore, studies highlighted that inconsistent referral criteria and service availability across geographical regions contributed to inequitable access to transitional care support (de Vries et al., 2019; Eyles et al., 2021; Jennings et al., 2020; Kupeli et al., 2019; Parker et al., 2021; Pond et al., 2019).

#### Medication management across transitions

Medication safety and continuity emerged as critical yet often overlooked components of effective care transitions for people with dementia. During hospital admissions, caregivers commonly voiced concerns about both under- and overmedication, particularly the use of sedatives and antipsychotics, which were seen to impair cognition and alertness (de Vries et al., 2019). Complex medication regimens were typical, and issues such as polypharmacy and inappropriate prescribing contributed to adverse drug events and unplanned hospital readmissions (Delgado et al., 2022; Gustafsson et al., 2018). These risks were exacerbated by inconsistent assessment of cognitive status during admission, which hindered accurate prescribing decisions (Gustafsson et al., 2018).

Medication-related problems frequently continued at discharge. Conversations with health care professionals were often rushed and focused on logistics rather than providing families with clear explanations of new or adjusted prescriptions, including potential side effects, treatment duration, or warning signs of adverse reactions (Sawan et al., 2021). Few caregivers received practical tools such as dose-decision aids to support safe medication management at home (Pond et al., 2019).

Transitions into hospital from home or care settings also offered missed opportunities for medication review and optimization (Kable et al., 2019). Services such as the Dementia Intensive Support model helped reduce avoidable transitions by addressing behavioral symptoms pharmacologically and reviewing physical health medications (Rubinsztein et al., 2020). Interdisciplinary approaches, particularly those involving pharmacists, were associated with improved outcomes through tailored medication lists and structured reconciliation processes (Gustafsson et al., 2018; Sawan et al., 2021).

Despite these promising interventions, studies highlighted persistent inequities. Compared to individuals without dementia, those with dementia were significantly less likely to receive accurate medication histories, comprehensive discharge education, or follow-up instructions (Prusaczyk et al., 2019). These disparities underscore the need for dementia-specific medication protocols to ensure safe, informed transitions across care settings.

#### Financial and policy constraints

Systemic financial and policy constraints often shaped the scope and equity of transition options for people living with dementia and their caregivers. In several studies, caregivers described how profit-driven care models, insurance limitations, or lack of subsidized services restricted choices and increased out-of-pocket costs during critical transitions (Armstrong et al., 2020; Cottrell et al., 2020; Fitzpatrick & Grace, 2019; Freedman et al., 2022; Gilmore-Bykovskyi et al., 2021; Gresham et al., 2018; Hanssen & Tran, 2019; Holton et al., 2023; Hui et al., 2022; Kupeli et al., 2019; Nguyen et al., 2022; Statz et al., 2022; Zmora et al., 2021). In contrast, more supportive policy environments, such as the Netherlands’ government-funded nursing homes or Medicaid alternatives in the United States, were reported to ease financial strain and promote smoother transitions into home or community-based care (Groenvynck et al., 2022; Harrison et al., 2023).

#### Systemic and structural facilitators and innovations supporting transitions

In contrast to the many structural barriers identified, several studies highlighted systemic innovations, such as supportive policies, governance frameworks, and funding reforms, alongside structural enablers like integrated service models and care coordination initiatives.

Proactive planning and clear documentation emerged as foundational supports for successful transitions. Advance Care Planning (ACP) tools, including Medical Orders for Scope of Treatment and Do Not Attempt Resuscitation (DNAR) forms, helped clarify preferences and reduce uncertainty around treatment and transition decisions (Hanson et al., 2019; Kupeli et al., 2019). Despite the terminal trajectory of dementia, palliative care consultations were infrequent, and many caregivers remained unaware of ACP processes, leaving them unprepared and distressed during critical care transition decisions (Mogan et al., 2022; Radcliffe et al., 2023; Saragosa et al., 2023; Sharda et al., 2020). Early and structured palliative care consultations played a critical role in aligning care with family goals, such as enabling people with dementia to remain at home until death (Chu et al., 2020; Hanson et al., 2019). Importantly, multiple studies advocated for these supports to be introduced earlier in the disease trajectory, not reserved solely for end-of-life scenarios (Brooks et al., 2022b; Cottrell et al., 2020; Freedman et al., 2022).

At the service delivery level, integrated care models were key enablers of smoother transitions. The Dementia Care Co-Management Model, for example, embedded care managers and comprehensive planning tools to proactively manage transitions and reduce avoidable hospital admissions (Jennings et al., 2020). Similarly, dedicated care coordinators, such as case managers or primary nurses, were found to guide families effectively through complex transitions (Khemai et al., 2022; Saragosa et al., 2023; Zmora et al., 2021). Specialist services, including Memory Clinics and Admiral nurses, also provided expert support tailored to the evolving needs of families during transition periods (Liu et al., 2021; Mogan et al., 2022).

Home-based primary care (HBPC) represented a particularly promising model that addressed continuity and coordination across the disease trajectory. Delivered by interdisciplinary teams directly in the home, HBPC reduced unnecessary hospitalizations and facilitated access to in-home palliative care. Compared to standard care, people receiving HBPC were more likely to die at home and access hospice services—outcomes highly valued by families. Caregivers reported that HBPC alleviated their burden by offering comprehensive, consistent support and reducing the need to navigate fragmented systems (Nguyen et al., 2022). Continuity in primary care also emerged as a key protective factor in transitional outcomes. Ongoing relationships with general practitioners built trust, promoted earlier identification of support needs, and were associated with lower rates of emergency hospitalization and delirium (Delgado et al., 2022; Godard-Sebillotte et al., 2021; Lei et al., 2020; Nguyen et al., 2022; Toles et al., 2022b; Zmora et al., 2021).

Unfortunately, persistent inequities in access to these models of care sometimes undermine these benefits. People from lower-income backgrounds and rural regions faced greater barriers to continuous primary care and specialist services (Brooks et al., 2022a; Eyles et al., 2021). Although telehealth was identified as a tool to bridge this gap, particularly after hospital discharge, rural residents were less likely to access hospitals offering such services, perpetuating geographic disparities (Wang et al., 2022a).

Well-designed structural factor innovations, including proactive care planning, integrated service delivery, and consistent primary care, play a vital role in easing transitions for people with dementia and their families. These approaches not only address structural gaps but also help reduce the overreliance on informal caregivers to coordinate complex, emotionally charged transitions.

## Theme 2: the role of the health and social care workforce in transitions

### How workforce skills, communication, and capacity shape transition experiences

Health care professionals were described as central figures in shaping the quality and emotional burden of care transitions. Their expertise, communication style, and availability significantly influenced how transitions were experienced by both people living with dementia and their informal caregivers (Wang et al., 2022b). Studies highlighted not only the clinical importance of skilled staff, but also the need for empathy, consistency, and effective information-sharing to support families throughout the transition journey.

#### Gaps in dementia-specific knowledge and training

Many caregivers highlighted a widespread lack of dementia-specific expertise across health care settings. Inadequate knowledge of dementia, particularly less common subtypes such as frontotemporal dementia and Lewy body dementia, contributed to uncertainty and inconsistent care during transitions (Armstrong et al., 2020; Rognstad et al., 2020). Knowledge gaps were especially prominent in end-of-life care, where staff often avoided conversations about death and dying despite their importance (Cronfalk et al., 2018; Mogan et al., 2022). Several studies recommended formal dementia education and end-of-life training, such as through the Gold Standards Framework, to improve staff confidence and competence (Aaltonen et al., 2014; Fitzpatrick & Grace, 2019; Freedman et al., 2022; Gresham et al., 2018; Rubinsztein et al., 2020; Saragosa et al., 2023; Zmora et al., 2021).

#### Empathy, communication, and staff attitudes

Beyond knowledge gaps, staff attitudes and communication styles played a central role in shaping transition experiences. When communication was poor, whether inconsistent, delayed, or overly technical, caregivers frequently felt isolated, overwhelmed, and excluded from decision making (Freedman et al., 2022; Gilmore-Bykovskyi et al., 2021; Holton et al., 2023). Caregivers reported confusion and frustration arising from conflicting advice, limited updates, and a lack of clarity about care plans (Armstrong et al., 2020; de Vries et al., 2019; Groenvynck et al., 2022; Khemai et al., 2022). These challenges were particularly evident during discharge, prognostic discussions, and the management of neuropsychiatric symptoms (Parker et al., 2021; Prusaczyk et al., 2020; Radcliffe et al., 2023; Sawan et al., 2021). Staff were also reported to sometimes overlook the cognitive limitations of people with dementia, such as by asking them for medication histories, leading to incomplete or inaccurate information (Ashbourne et al., 2021).

In contrast, when communication was timely, clear, and adapted to the needs of families, transitions were significantly improved. Multimodal communication (e.g., phone calls, emails, face-to-face meetings) and regular updates from health care professionals, especially nurses and psychologists, helped caregivers feel informed, reassured, and more engaged in the care process (Marsh et al., 2023; Rognstad et al., 2020). Caregivers consistently valued staff who were emotionally supportive, responsive, and proactive in involving them in care planning (Khemai et al., 2022; Zmora et al., 2021). Respectful and compassionate care was repeatedly described as a key facilitator of smoother, more person-centered transitions (Zmora et al., 2021).

Importantly, when health care professionals recommended a care transition themselves, it sometimes relieved the emotional burden for families, helping to validate their decisions and reduce feelings of guilt (Wright et al., 2024). Some caregivers described the ideal support figure as a “cheerleader,” a trusted individual who was clinically competent, empathetic, and consistently available throughout the transition journey (Saragosa et al., 2023).

#### Staffing pressures and continuity challenges

A major workforce barrier affecting transitions was the availability of staff. Chronic understaffing in care facilities undermined continuity of care and created anxiety for families (Armstrong et al., 2020; Brooks et al., 2022b; Rognstad et al., 2020; Smith & Phillipson, 2022; Tropea et al., 2022). High staff turnover made it difficult to build trusting relationships, essential for personalized dementia care (Khemai et al., 2022; Mogan et al., 2022). Caregivers linked these workforce issues to broader organizational pressures. Reports on overburdened staff, limited time, and insufficient resources were common, with the resulting strain negatively affecting the quality of care and communication during transitions (de Vries et al., 2019). Without adequate staffing and continuity, the ability to deliver thoughtful, person-centered care was diminished, increasing caregiver distress and complicating the transition process.

## Theme 3: caregiving, emotions, and decision making in transitions

### How informal caregivers experience and influence transitions, and the emotional consequences

Caregivers play a pivotal role in supporting people with dementia during care transitions. Their knowledge, emotional readiness, and decision-making capacity significantly shape the transition experience. Across studies, caregivers reported challenges related to timing, emotional strain, knowledge gaps, advocacy efforts, and the complexities of choosing appropriate care settings. These issues were often compounded by a lack of systemic and structural support, peer guidance, and personalized assistance.

#### Knowledge and support networks

Caregivers frequently expressed a lack of dementia-specific knowledge, which hindered their ability to anticipate care needs and make informed decisions during transitions (de Vries et al., 2019; Ke-Hau et al., 2020; Leggett et al., 2018). Education on disease progression and practical coping strategies were identified as key needs (Brooks et al., 2022b; Damien et al., 2020). When present, dementia education and respite services reduced stress and enabled caregivers to make timely transitions to long-term care (Gresham et al., 2018).

Support from informal networks also played a critical role. Studies highlighted the value of peer support, transitional counselling, and buddy programs, which helped caregivers feel less isolated and more confident in their decision making around care transitions (Brooks et al., 2022b; Johannessen et al., 2019; Rubinsztein et al., 2020). Conversely, inconsistent or absent social support exacerbated grief, guilt, and uncertainty (Statz et al., 2022). Affirmations from others and reflective practices such as diary-keeping helped caregivers reaffirm the necessity of transitions (Wright et al., 2024).

#### Caregiver advocacy and role recognition during care transitions

Caregivers frequently maintained an active role in care, even after transitions to hospital or long-term care. This involvement was driven by a desire to advocate for loved ones, oversee care plans, and preserve personal routines (Cottrell et al., 2020; Cronfalk et al., 2018). During hospital admissions, caregivers often felt a duty to remain present, especially when the person with dementia could not communicate their needs (de Vries et al., 2019; Parker et al., 2021).

However, caregivers also reported frequent exclusion from decision making and inconsistencies in how their role was recognized by professionals (Marsh et al., 2023). Advocacy efforts were emotionally and physically demanding, particularly in emergency settings where long waits and lack of information were common (de Vries et al., 2019). Despite these barriers, caregiver involvement was linked to smoother transitions and improved outcomes for people with dementia (Khemai et al., 2022).

#### Emotions about timing and decision making

A central theme in caregiver narratives was the emotional complexity of determining when and how to transition to higher levels of care. Families often described a sense of “waiting in limbo,” gradually providing more support until a crisis necessitated formal care (Cronfalk et al., 2018). Early diagnosis and proactive counselling about disease trajectory enabled families to plan, reducing stress and enhancing decision confidence (Brooks et al., 2022b; Groenvynck et al., 2022; van der Heide et al., 2021).

Poorly timed or unplanned transitions, such as emergency hospital admissions or rushed discharges, intensified anxiety and undermined caregivers’ sense of control (Sawan et al., 2021; Wright et al., 2024). Emotional responses to transitions for both the individual with dementia and caregivers ranged from guilt and grief to relief and acceptance, depending on the level of preparation and perceived quality of care (Fitzpatrick & Grace, 2019; Statz et al., 2022). Involving the person with dementia in the decision, where possible, was associated with greater satisfaction and lower distress (Davison et al., 2019).

#### Navigating facility selection and residential transitions

Selecting a care facility was a unique stressor distinct from broader systemic and structural navigation challenges. Caregivers often described the process as overwhelming, shaped by financial constraints, limited availability, and urgency to accept the first available option (Brooks et al., 2022b; Rapp et al., 2018; Rognstad et al., 2020). Inadequate support for this process, especially regarding respite care, frequently led to exhaustion and guilt (Gilmore-Bykovskyi et al., 2021; Wright et al., 2024). Concerns around care quality, safety, and dignity influenced placement decisions. Caregivers reported distress over depersonalized or unsafe environments and preferred facilities with person-centered approaches that incorporated personal histories and belongings (Smith et al., 2023; Zmora et al., 2021).

Support from care placement consultants and purpose-built memory support units facilitated better outcomes (Broadbent & Gilbert, 2020; Radcliffe et al., 2023). Multidisciplinary teams and dementia-integrated care models in assisted living, skilled nursing settings, and cottage models of respite care were also positively viewed (Gresham et al., 2018; Harkin et al., 2020; Johannessen et al., 2019; Manis et al., 2021).

Together, these findings underscore the deeply emotional, practical, and relational dimensions of caregiving during transitions. Strengthening caregiver support, ensuring timely guidance, and recognizing advocacy roles are critical to improving transition experiences for both families and people living with dementia.

#### Emotional adjustment, conflict, and identity

Care transitions, particularly into long-term care, often triggered complex emotional responses for both caregivers and people living with dementia. The decision to move a loved one, frequently perceived as a “last resort,” was commonly met with resistance, anxiety, and grief (Fitzpatrick & Grace, 2019; Hui et al., 2022; Saragosa et al., 2023; Wright et al., 2024). Caregivers described difficulty initiating the conversation about relocation and noted that individuals with dementia sometimes responded with anger, distress, or confusion (Davison et al., 2019; Rognstad et al., 2020). These tensions occasionally required urgent intervention and placed further strain on caregiver–recipient relationships.

After the move, caregivers often reported feelings of powerlessness, describing how health and social care systems appeared to make final decisions without their input (Mogan et al., 2022; Rognstad et al., 2020). Many expressed a sense of lost autonomy, identity disruption, or “failure,” particularly when the person with dementia was their spouse or parent (Ashbourne et al., 2021; Brooks et al., 2022b; Holton et al., 2023). Long-term grief, loneliness, and even clinical depression were described, with some caregivers seeking professional support well after the transition (Brooks et al., 2022a; Hähnel et al., 2023).

People living with dementia also experienced emotional consequences, reporting loss of independence, disconnection from community, and diminished quality of life when transitions were poorly managed (Davison et al., 2019; Johannessen et al., 2019). Over time, some caregivers and individuals adapted to the new care environment, reporting relief, safety, and improved well-being. Acceptance was more likely when transitions were planned collaboratively and the person’s wishes were acknowledged (Ashbourne et al., 2021; Fekonja et al., 2021).

## Theme 4: cultural, social, and situational influences on transition pathways

### How cultural values influence transition decisions and experiences

Care transitions for people with dementia are shaped not only by structural and systemic factors but also by deeply personal, cultural, and contextual dynamics. Across studies, the beliefs, resources, and circumstances of families, such as cultural norms, rurality, dementia subtype, and socioeconomic status, significantly influenced how transitions were understood, experienced, and navigated. This theme highlights how diverse identities and environments shaped caregiving expectations, access to services, and emotional responses to transitions.

#### Cultural beliefs, stigma, and moral expectations

Caregivers often faced internal and external pressures rooted in cultural expectations around family caregiving, particularly when considering transitions to long-term care. In collectivist cultures, the moral obligation to care for aging parents at home created strong resistance to institutional care, even when caregiving demands became overwhelming (Fekonja et al., 2021; Hanssen et al., 2022; Hanssen & Tran, 2019; Holton et al., 2023; Hui et al., 2022). Feelings of guilt, shame, and perceived failure were frequently reported, especially when caregivers were influenced by negative societal judgments or the disapproval of other family members (Johannessen et al., 2019; Statz et al., 2022).

Families described a sense of failure both in relinquishing day-to-day care and in being unable to shield loved ones from institutional environments they felt were not always respectful or safe (de Vries et al., 2019; Fekonja et al., 2021). This emotional toll was often compounded by a lack of culturally sensitive support services. Studies found that when transitions were facilitated in ways that respected cultural values and helped families navigate stigma, caregivers experienced more acceptance and less distress (Aaltonen et al., 2014; Hanson et al., 2019; Hanssen et al., 2022).

## Discussion and implications

This review synthesized 67 studies examining care transitions for people with dementia, highlighting persistent structural, systemic, interpersonal, and experiential challenges. Transitions were often experienced as emotionally complex, disorienting, and stressful for both people living with dementia and their caregivers, who frequently faced uncertainty, fragmented support, and high decision-making burdens. Findings revealed that transitions are hindered by fragmented health care systems, inadequate communication, limited dementia-specific knowledge among professionals, and heavy reliance on informal caregivers. Despite these barriers, promising innovations, including proactive care planning, integrated care models, and home-based services, demonstrated potential to improve continuity and person-centeredness in transitional care.

These findings underscore the complexity of care transitions for people with dementia and their caregivers, where systemic and structural factors, and interpersonal dynamics intersect to shape outcomes. The persistence of structural health and social care fragmentation and information gaps suggests that existing health care systems remain poorly equipped to meet the needs of this population, despite long-standing recognition of these issues in the literature (Giebel et al., 2021; Martin et al., 2020). Previous studies have similarly highlighted the negative impact of disjointed care and limited continuity across settings (Britton & Zimmermann, 2022; Smith et al., 2021), yet this present synthesis reveals that such problems continue to affect people with dementia and their families. This disconnect may reflect ongoing implementation gaps between policy ambitions and frontline realities, where integrated, person-centered care remains aspirational due to insufficient resourcing, leadership, and system alignment. Moreover, entrenched fragmentation, driven by siloed services, incompatible systems, and differing professional mandates, appears to continue to impede collaboration and continuity during critical transitions. Compounding these challenges is the ongoing lack of dementia-specific training among health and social care professionals, despite the availability of evidence-based frameworks to improve care competencies (Gkioka et al., 2020; Surr et al., 2020). Without adequate training, protected time, and structural support to work across disciplines, staff are often ill-equipped to deliver coordinated, dementia-informed care, particularly in high-pressure transition points where communication and clinical judgment are critical.

Notably, our review extends prior work by emphasizing the emotional and moral weight of transition decisions, especially among caregivers navigating cultural expectations and limited formal support. While some earlier studies have explored caregiver burden (Germain et al., 2024; Groenvynck et al., 2022), few have examined how feelings of guilt, failure, and cultural stigma interact with dementia transition pathways. The identification of emerging models, such as ACP, contributes to the growing evidence base demonstrating that these innovations can reduce distress and better align care with the preferences of people with dementia and their families (Wendrich-van Dael et al., 2020). Similarly, models like HBPC are gaining increasing recognition for their potential to enhance continuity and person-centeredness in transitional care (Li et al., 2022; Sy et al., 2024; Weiner et al., 2024). Unfortunately, these benefits appear unevenly distributed, with continued inequities linked to geography, socioeconomic status, and service availability (Giebel et al., 2025; Watson et al., 2021). Individuals with rarer forms of dementia may face even greater challenges in accessing appropriate transitional support, due to lower clinical awareness and limited availability of tailored services (Giebel et al., 2025).

## Strengths and limitations

This review offers a comprehensive and contemporary synthesis of the evidence on care transitions for people living with dementia and their caregivers, drawing on a diverse range of study designs and highlighting systemic, structural and interpersonal factors that shape transition experiences. A key strength was that no restrictions were placed on the country of origin of included studies, as incorporating research from diverse international settings allowed for a more comprehensive synthesis of how varying health care systems and policy contexts shape dementia-related care transitions. This international scope, combined with a thematic synthesis approach, enabled the integration of findings across qualitative and quantitative studies, capturing both systemic and structural dynamics and the lived experiences of caregivers and, to a lesser extent, people with dementia.

However, several limitations should be acknowledged. As translation resources were not available, the review was limited to English-language publications, which may have resulted in the exclusion of relevant studies published in other languages. The predominance of studies conducted in high-income, Western countries further limits the generalizability of findings to other cultural contexts and health system structures. Additionally, the evidence base relied heavily on carer perspectives; the voices of people living with dementia, especially those with advanced dementia, were underrepresented. Most studies focused on transitions into long-term care, with comparatively little attention to transitions between hospital and home, or between community-based services. This concentration on long-term care may be attributable to several factors, including the relative predictability and visibility of these transitions, their association with significant policy and cost implications, and the feasibility of participant recruitment and data collection. In contrast, transitions such as acute hospitalizations or respite care are often less systematically documented, more variable in nature, and methodologically challenging to study. These gaps underscore an important area for future research to better understand and support the diverse transition experiences of people living with dementia.

This review highlights the urgent need for more integrated, equitable, and person-centered approaches to dementia-related care transitions. Despite policy commitments to improve continuity and coordination, persistent barriers—such as fragmented systems, variable workforce competencies, and limited access to tailored support—continue to hinder effective transitional care. The findings reinforce the critical role of informal caregivers, whose experiences and emotional labor remain central to navigating complex systems. Emerging models such as HBPC and structured ACP offer promising pathways forward, but current access to these interventions is uneven and often dependent on geography, diagnosis subtype, and socioeconomic status. To address these disparities and improve outcomes, future policy and service development must prioritize the inclusion of underrepresented populations, investment in workforce training, and support for a wider range of transitions across the care continuum. Advancing this agenda will require sustained cross-sector collaboration, implementation research, and meaningful engagement with both caregivers and people living with dementia.

## Supplementary Material

gnaf275_Supplementary_Data

## Figures and Tables

**Table:** 

Task	Co-author contribution
**Conception and design**	E.Bu., B.C.M.S., L.G., D.H.
**Search strategy**	L.G., E.Bu., B.C.M.S., J.B. (University of Curtin librarian)
**Title and abstract sifting**	L.G., J.B., A.H.K., S.S.
**Full-text review**	L.G., J.B., A.H.K, E.Bu., C.M., E.B., K.H., A.S., D.H., B.B., S.S., B.C.M.S., B.M.
**Critical appraisal**	L.G., J.B., C.M., K.H., S.S., E.Y.H.T., A.H.K., A.S., E.Bu., P.W.
**Data extraction**	LG, P.W., J.B.
**Manuscript writing**	L.G.
**Revision**	All authors

## Data Availability

All data supporting the findings of this study are available within the article and its supplementary/appendix files. Further enquiries can be directed to the corresponding author. This review has been registered on PROSPERO: CRD42023452669.
