# Supplementary material for: Mind the gap: a systematic review of barriers, facilitators, and experiences of care transitions for people living with dementia and their informal caregivers
Source: Gerontologist. 2025 Nov 25;66(1):gnaf275. doi: 10.1093/geront/gnaf275 (PMC12854086; doi:10.1093/geront/gnaf275)
Supplement: gnaf275_Supplementary_Data [file gnaf275_supplementary_data.pdf]

Supplementary Material

Appendix A. PRISMA checklist.

Section and Topic	Item #	Checklist item	Location where item is reported (page numbers)
**TITLE**			
Title	1	Identify the report as a systematic review.	1
**ABSTRACT**			
Abstract	2	See the PRISMA 2020 for Abstracts checklist.	1
**INTRODUCTION**			
Rationale	3	Describe the rationale for the review in the context of existing knowledge.	2
Objectives	4	Provide an explicit statement of the objective(s) or question(s) the review addresses.	2
**METHODS**			
Eligibility criteria	5	Specify the inclusion and exclusion criteria for the review and how studies were grouped for the syntheses.	3-4
Information sources	6	Specify all databases, registers, websites, organisations, reference lists and other sources searched or consulted to identify studies. Specify the date when each source was last searched or consulted.	3
Search strategy	7	Present the full search strategies for all databases, registers and websites, including any filters and limits used.	Appendix B
Selection process	8	Specify the methods used to decide whether a study met the inclusion criteria of the review, including how many reviewers screened each record and each report retrieved, whether they worked independently, and if applicable, details of automation tools used in the process.	4-5
Data collection process	9	Specify the methods used to collect data from reports, including how many reviewers collected data from each report, whether they worked independently, any processes for obtaining or confirming data from study investigators, and if applicable, details of automation tools used in the process.	4
Data items	10a	List and define all outcomes for which data were sought. Specify whether all results that were compatible with each outcome domain in each study were sought (e.g.	3-4

Section and Topic	Item #	Checklist item	Location where item is reported (page numbers)
		for all measures, time points, analyses), and if not, the methods used to decide which results to collect.	
	10b	List and define all other variables for which data were sought (e.g. participant and intervention characteristics, funding sources). Describe any assumptions made about any missing or unclear information.	Supplementary File 1
Study risk of bias assessment	11	Specify the methods used to assess risk of bias in the included studies, including details of the tool(s) used, how many reviewers assessed each study and whether they worked independently, and if applicable, details of automation tools used in the process.	5 and Appendix C
Effect measures	12	Specify for each outcome the effect measure(s) (e.g. risk ratio, mean difference) used in the synthesis or presentation of results.	N/A
Synthesis methods	13a	Describe the processes used to decide which studies were eligible for each synthesis (e.g. tabulating the study intervention characteristics and comparing against the planned groups for each synthesis (item #5)).	3-4
	13b	Describe any methods required to prepare the data for presentation or synthesis, such as handling of missing summary statistics, or data conversions.	N/A
	13c	Describe any methods used to tabulate or visually display results of individual studies and syntheses.	Supplementary File 1
	13d	Describe any methods used to synthesize results and provide a rationale for the choice(s). If meta-analysis was performed, describe the model(s), method(s) to identify the presence and extent of statistical heterogeneity, and software package(s) used.	4
	13e	Describe any methods used to explore possible causes of heterogeneity among study results (e.g. subgroup analysis, meta-regression).	N/A
	13f	Describe any sensitivity analyses conducted to assess robustness of the synthesized results.	N/A
Reporting bias assessment	14	Describe any methods used to assess risk of bias due to missing results in a synthesis (arising from reporting biases).	N/A

Section and Topic	Item #	Checklist item	Location where item is reported (page numbers)
Certainty assessment	15	Describe any methods used to assess certainty (or confidence) in the body of evidence for an outcome.	N/A
**RESULTS**			
Study selection	16a	Describe the results of the search and selection process, from the number of records identified in the search to the number of studies included in the review, ideally using a flow diagram.	Figure 1
	16b	Cite studies that might appear to meet the inclusion criteria, but which were excluded, and explain why they were excluded.	N/A
Study characteristics	17	Cite each included study and present its characteristics.	Supplementary File 1
Risk of bias in studies	18	Present assessments of risk of bias for each included study.	Appendix C
Results of individual studies	19	For all outcomes, present, for each study: (a) summary statistics for each group (where appropriate) and (b) an effect estimate and its precision (e.g. confidence/credible interval), ideally using structured tables or plots.	N/A
Results of syntheses	20a	For each synthesis, briefly summarise the characteristics and risk of bias among contributing studies.	Supplementary File 1 and Appendix C
	20b	Present results of all statistical syntheses conducted. If meta-analysis was done, present for each the summary estimate and its precision (e.g. confidence/credible interval) and measures of statistical heterogeneity. If comparing groups, describe the direction of the effect.	N/A
	20c	Present results of all investigations of possible causes of heterogeneity among study results.	N/A
	20d	Present results of all sensitivity analyses conducted to assess the robustness of the synthesized results.	N/A
Reporting biases	21	Present assessments of risk of bias due to missing results (arising from reporting biases) for each synthesis assessed.	N/A
Certainty of evidence	22	Present assessments of certainty (or confidence) in the body of evidence for each outcome assessed.	N/A

Section and Topic	Item #	Checklist item	Location where item is reported (page numbers)
**DISCUSSION**			
Discussion	23a	Provide a general interpretation of the results in the context of other evidence.	15-18
	23b	Discuss any limitations of the evidence included in the review.	15-18
	23c	Discuss any limitations of the review processes used.	18
	23d	Discuss implications of the results for practice, policy, and future research.	18
**OTHER INFORMATION**			
Registration and protocol	24a	Provide registration information for the review, including register name and registration number, or state that the review was not registered.	1 and 3
	24b	Indicate where the review protocol can be accessed, or state that a protocol was not prepared.	1 and 3
	24c	Describe and explain any amendments to information provided at registration or in the protocol.	N/A
Support	25	Describe sources of financial or non-financial support for the review, and the role of the funders or sponsors in the review.	Title page
Competing interests	26	Declare any competing interests of review authors.	Title page
Availability of data, code and other materials	27	Report which of the following are publicly available and where they can be found: template data collection forms; data extracted from included studies; data used for all analyses; analytic code; any other materials used in the review.	Title page

Appendix B. Search strategy

	CONCEPT 1	CONCEPT 2	CONCEPT 3[1]
**Keywords**	(dement* or alzheim*)<br><br>Title/abstract	(transition* or transfer* or admission or relocat* or placement* or resettl* or "care coordination" or "hospital to home" or "hospital discharge" or "continuity of care" or "hand-off" or handoff or "hand-over" or handover or "post discharge" or "follow-up" or followup or "hospitali?ation" or "institutionali?ation" or "care assessment")<br><br>Title/abstract	("acute care" or "aged care" or "assisted living" or "community care" or "home care" or "long-term care" or ambula* or hospital* or rehab* or "nursing home" or "residential care" or "respite care" or "palliative care" or "elder* care" or "hospice care" or respite or institution* or "fragmented care" or "planned care")<br><br>OR<br><br>(geriatric adj2 (home* or facilit* or institution* or care))<br><br>Title/abstract
	**Subject Headings**		
**Medline (Mesh)**	exp Dementia/	Continuity of Patient Care/ or Aftercare/ or Hospital to Home Transition/ or Patient Discharge/ or Patient Handoff/ or Patient Transfer/ or Transitional Care/	Homes for the Aged/ or Assisted Living Facilities/ or exp Nursing Homes/<br><br>Hospitals/Emergency Service, Hospital/Critical Care/Intensive Care Units/Ambulatory Care Facilities/<br><br>Adult Day Care Centers/Hospice Care/Palliative Care/<br><br>Home Care Services
**Embase**	exp dementia/	patient care/ or hospital discharge/ or hospital to	residential home/assisted living/nursing home/

[1] Concept 3 was included in the search strategy as a deliberate and pragmatic measure to enhance the specificity of results while retaining adequate breadth. Despite its inclusion, the initial MEDLINE search yielded over 28,000 records, highlighting the need to focus the search on studies situated within relevant care settings. This concept was designed to encompass a broad range of healthcare environments, including acute, community, long-term, home-based, and palliative care, to ensure comprehensive coverage of the transitions experienced by people living with dementia and their informal caregivers.

		home transition/ or patient transport/ or clinical handover/ or transitional care/ or aftercare/	hospital/emergency health service/intensive care/<br>critical care/ambulatory care/<br><br>elderly day care/hospice care/palliative therapy/<br>home care/
**PsycInfo**	exp Dementia/	Continuum of Care/ or exp Facility Discharge/ or Aftercare/ or Discharge Planning/ or Client Transfer/	hospitalization<br>long term care<br>residential care<br>home care<br>palliative care<br>hospice<br>rehabilitation<br>community services
**CINAHL**	MH=<br>"Dementia+"	MH=<br>"Continuity of Patient Care" or "Patient Discharge" or "Early Patient Discharge" or "Discharge Planning" or "Transfer, Discharge" or "Hospital to Home Transition" or "Hand Off (Patient Safety)" or "Transitional Care" or "After Care"	MH = "Nursing Homes+" or "Housing for Older Persons" or "Assisted Living" or<br>"Residential Facilities+" or "Long-Term Care+" or "Day Care, Adult" or<br>"Home Health Care+" or "Community Health Services+" or "Hospitalization+" or "Acute Care" or "Emergency Services+" or<br>"Intensive Care Units+" or "Ambulatory Care+" or "Palliative Care+" or "Hospice Care+" or "Rehabilitation+"
**WoS**	Keywords only – title/abstract		
**Proquest Central**	Keywords only – title/abstract<br><br>Limit – Scholarly Journals		

Appendix C. JBI critical appraisal.

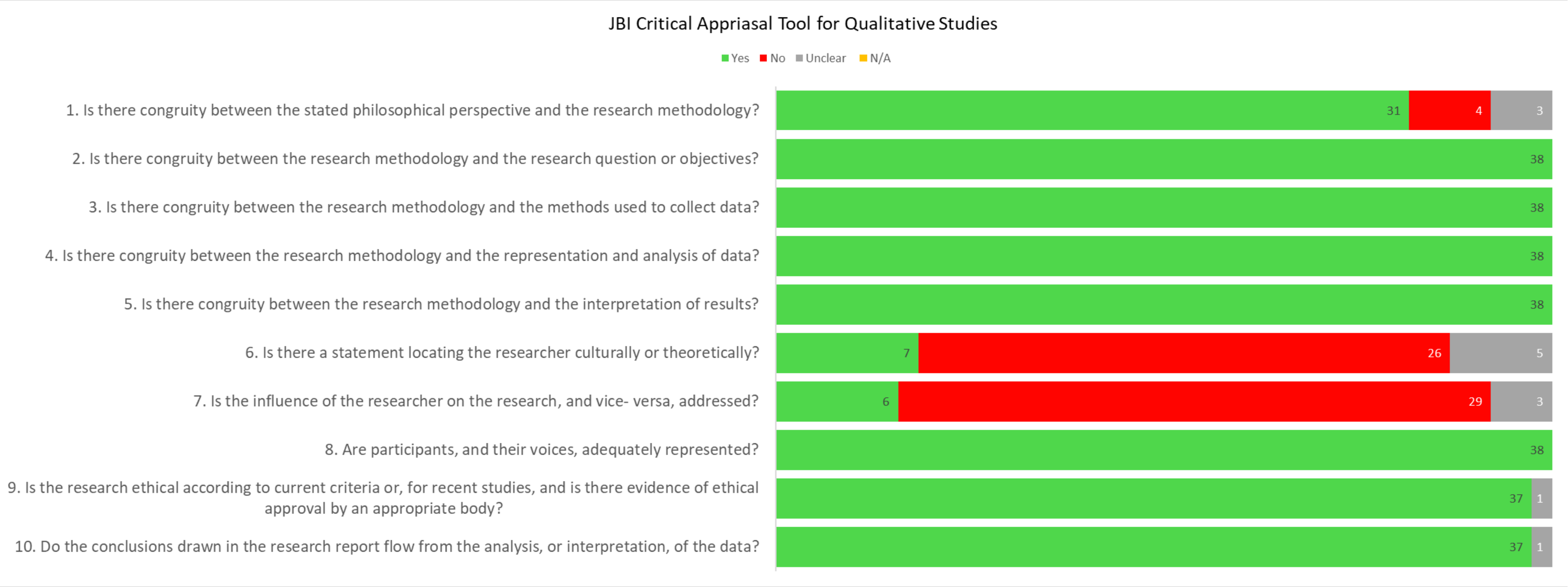

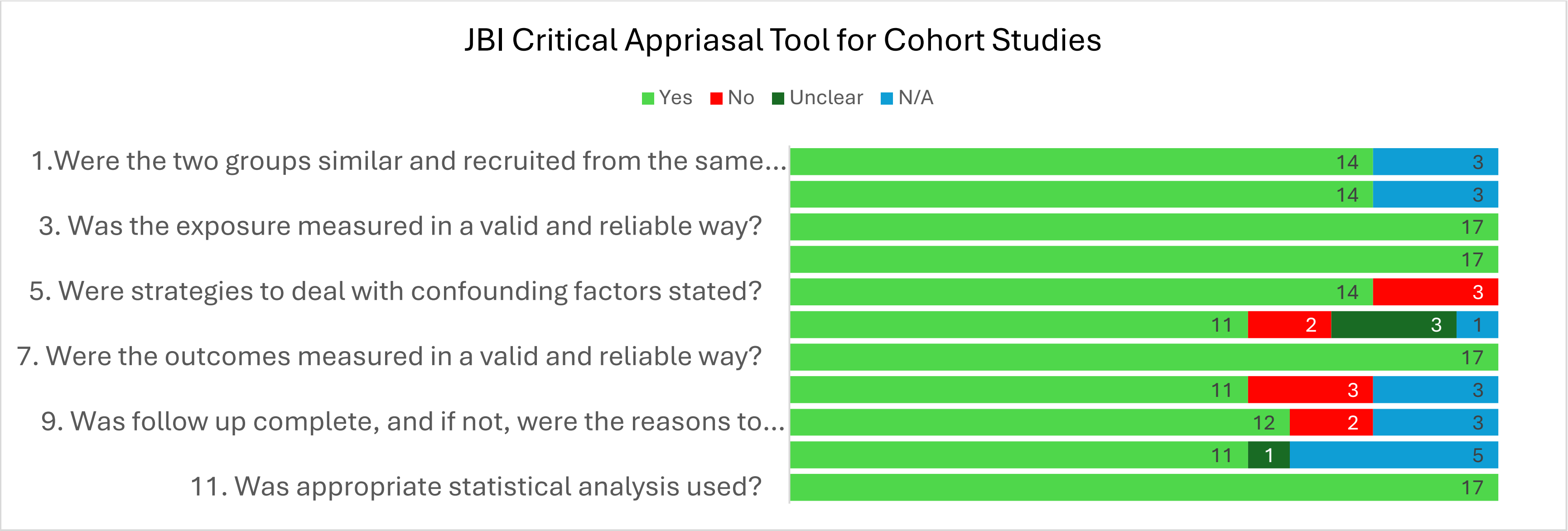
JBI Critical Appriasal Tool for Cohort Studies
Yes
No
Unclear
N/A
1.Were the two groups similar and recruited from the same...
3. Was the exposure measured in a valid and reliable way?
5. Were strategies to deal with confounding factors stated?
7. Were the outcomes measured in a valid and reliable way?
9. Was follow up complete, and if not, were the reasons to...
11. Was appropriate statistical analysis used?
14 3
14 3
17
17
14 3
11 2 3 1
17
11 3 3
12 2 3
11 1 5
17

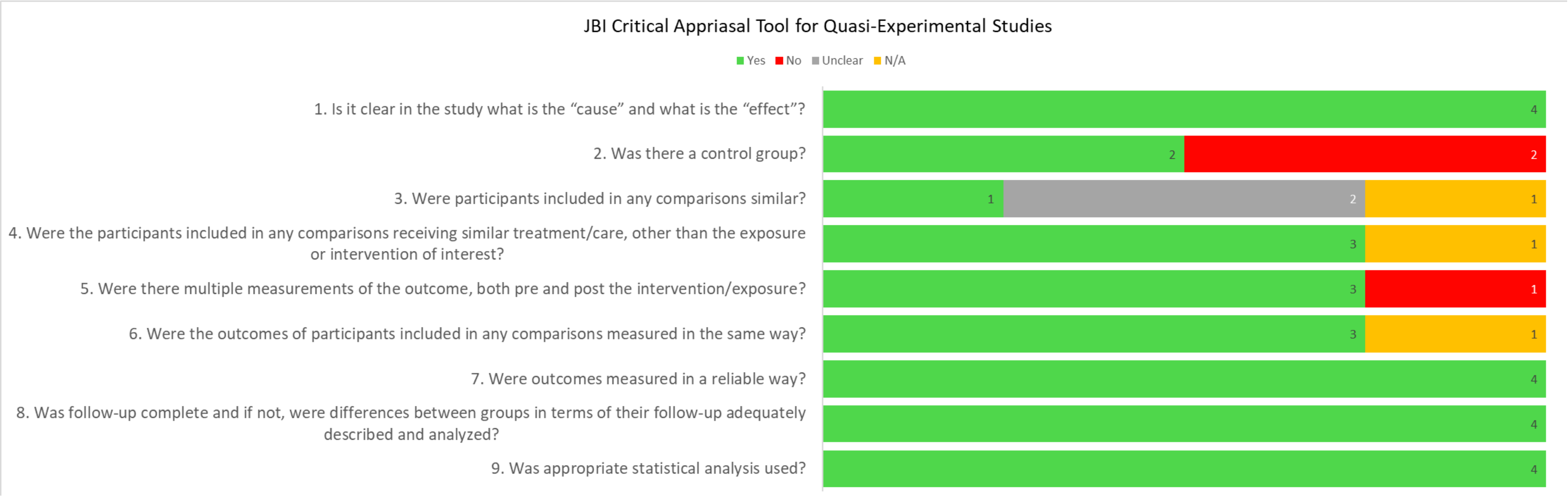
JBI Critical Appriasal Tool for Quasi-Experimental Studies
Yes
No
Unclear
N/A
1. Is it clear in the study what is the “cause” and what is the “effect”?
2. Was there a control group?
3. Were participants included in any comparisons similar?
4. Were the participants included in any comparisons receiving similar treatment/care, other than the exposure or intervention of interest?
5. Were there multiple measurements of the outcome, both pre and post the intervention/exposure?
6. Were the outcomes of participants included in any comparisons measured in the same way?
7. Were outcomes measured in a reliable way?
8. Was follow-up complete and if not, were differences between groups in terms of their follow-up adequately described and analyzed?
9. Was appropriate statistical analysis used?
4
2
2
1
2
1
3
1
3
1
3
1
4
4
4

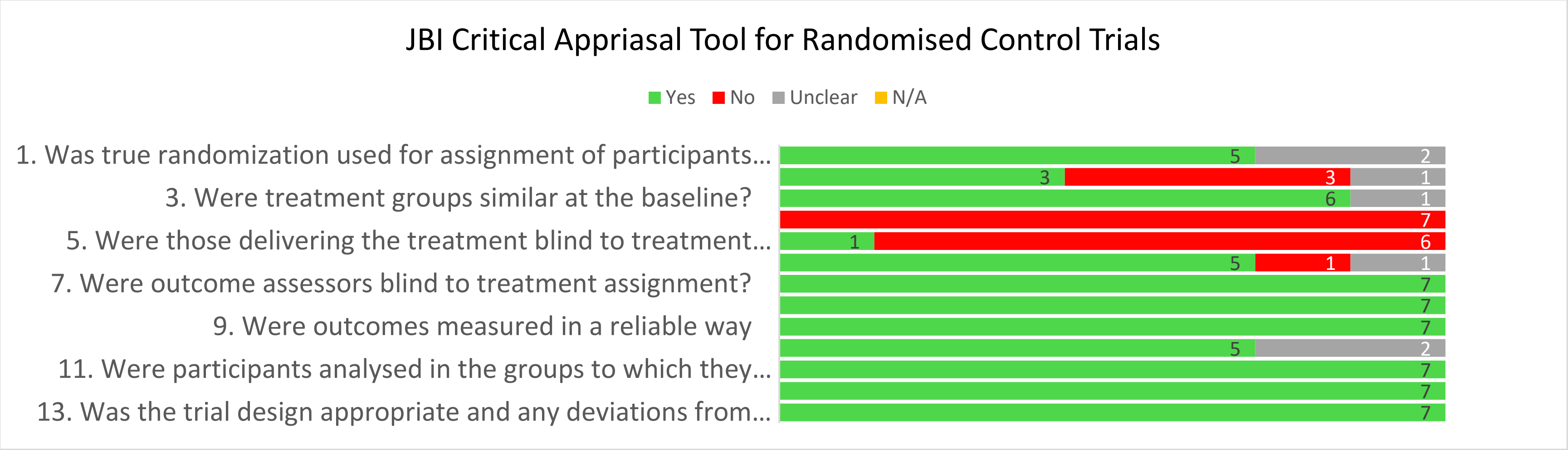
JBI Critical Appriasal Tool for Randomised Control Trials
Yes
No
Unclear
N/A
1. Was true randomization used for assignment of participants...
3. Were treatment groups similar at the baseline?
5. Were those delivering the treatment blind to treatment...
7. Were outcome assessors blind to treatment assignment?
9. Were outcomes measured in a reliable way
11. Were participants analysed in the groups to which they...
13. Was the trial design appropriate and any deviations from...
5 2
3 3 1
6 1
7
1 6
5 1 1
7
7
7
5 2
7
7
7

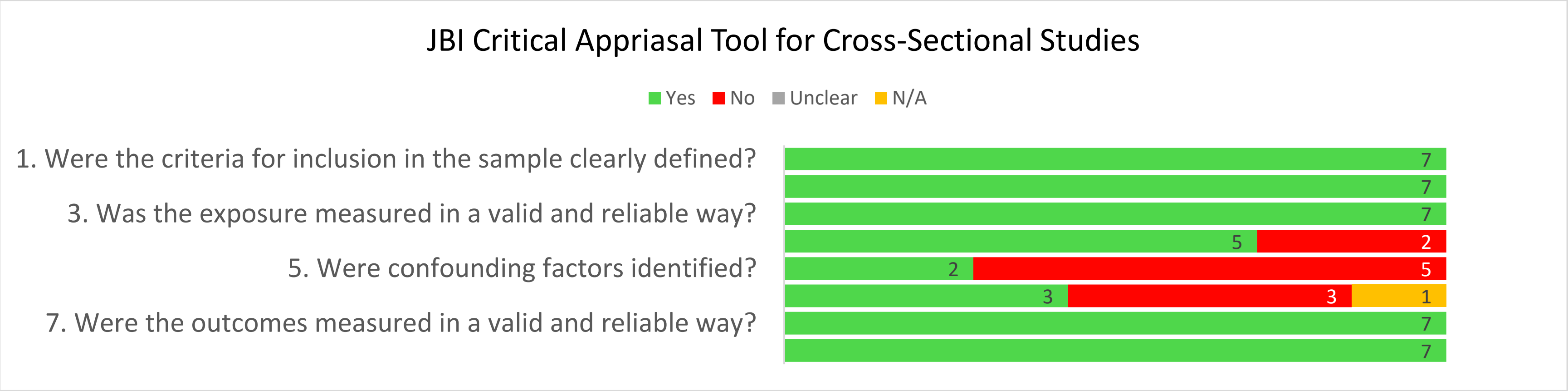
JBI Critical Appriasal Tool for Cross-Sectional Studies
Yes
No
Unclear
N/A
1. Were the criteria for inclusion in the sample clearly defined?
3. Was the exposure measured in a valid and reliable way?
5. Were confounding factors identified?
7. Were the outcomes measured in a valid and reliable way?
7
7
7
5 2
2 5
3 3 1
7
7

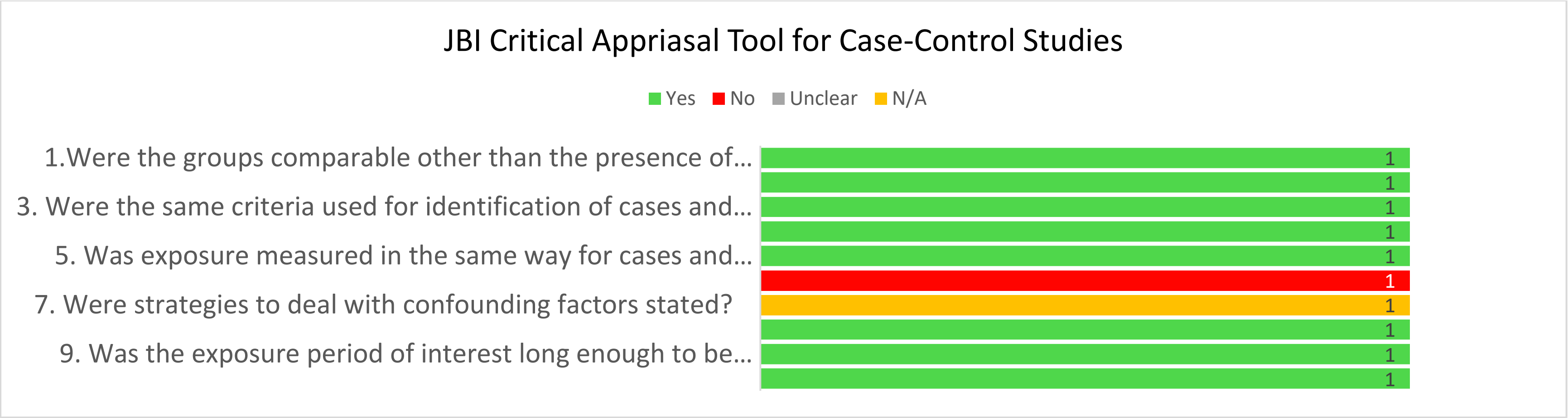
JBI Critical Appriasal Tool for Case-Control Studies
Yes
No
Unclear
N/A
1.Were the groups comparable other than the presence of...
3. Were the same criteria used for identification of cases and...
5. Was exposure measured in the same way for cases and...
7. Were strategies to deal with confounding factors stated?
9. Was the exposure period of interest long enough to be...
1
1
1
1
1
1
1
1
1
1

Appendix D. A summary of the number of studies that reported on barriers and facilitators.

Theme	Number of studies	References	Barriers	Facilitators
**Systemic and structural influences on transitions**	49	(Armstrong et al., 2020)<br>(Ashbourne et al., 2021)<br>(Brooks et al., 2022a)<br>(Brooks et al., 2022b)<br>(de Vries et al., 2019)<br>(Chu et al., 2020)<br>(Cottrell et al., 2020)<br>(Delgado et al., 2022)<br>(Eyles et al., 2021)<br>(Fekonja et al., 2021)<br>(Fitzpatrick & Grace, 2019)<br>(Freedman et al., 2022)<br>(Gilmore-Bykovskyi et al., 2021)<br>(Godard-Sebillotte et al., 2021)<br>(Gresham et al., 2018)<br>(Groenvynck et al., 2022)<br>(Gustafsson et al., 2018)<br>(Hanson et al., 2019)<br>(Hanssen & Tran, 2019)<br>(Harrison et al., 2023)<br>(Holton et al., 2023)<br>(Hui et al., 2022)<br>(Jennings et al., 2020)<br>(Johannessen et al., 2019)<br>(Kable et al., 2019)<br>(Khemai et al., 2022)	**1.1 Health system complexity and fragmentation:**<br>The operationalization of healthcare and policy systems is complex and varies across regions, creating confusion for caregivers and inconsistent care experiences. Fragmented care occurs due to poor integration between electronic health records, healthcare teams, and service providers. Individuals from rural or lower socioeconomic backgrounds face greater barriers to accessing care, including difficulty securing timely primary care appointments.<br><br>**1.2 Information gaps and system navigation challenges:**<br>Inconsistent, delayed, or overly technical communication from healthcare staff hindered transitions and left caregivers feeling overlooked. Discharge documentation was often lengthy, unclear, or failed to anticipate future	**1.1 Health system complexity and fragmentation:**<br>Systemic reforms that simplify administrative and referral processes improve care navigation. Integrated service delivery models and interoperable health records enhance communication across care settings. Government-supported programs that promote coordination and continuity of care, such as shared-care arrangements, reduce fragmentation.<br><br>**1.2 Information gaps and system navigation challenges:**<br>Smooth transitions were supported by clear, multimodal communication methods such as phone calls, emails, and face-to-face meetings. Regular interdisciplinary meetings helped anticipate needs and prevent complications. Advance Care Planning (ACP) tools and forms, including Medical Orders for Scope of Treatment (MOST), clarified care preferences and

		(Kupeli et al., 2019)<br>(Larsen et al., 2020)<br>(Leggett et al., 2018)<br>(Lei et al., 2020)<br>(Marsh et al., 2023)<br>(Mogan et al., 2022)<br>(Nguyen et al., 2022)<br>(Parker et al., 2021)<br>(Pocock et al., 2018)<br>(Pond et al., 2019)<br>(Prusaczyk et al., 2020)<br>(Radcliffe et al., 2023)<br>(Rognstad et al., 2020)<br>(Rubinsztein et al., 2020)<br>(Ryvicker et al., 2022)<br>(Saragosa et al., 2023)<br>(Sawan et al., 2021)<br>(Sharda et al., 2020)<br>(Smith & Phillipson, 2022)<br>(Statz et al., 2022)<br>(Toles et al., 2022a)<br>(Toles et al., 2022b)<br>(Wang et al., 2022a)<br>(Zmora et al., 2021)	care needs, contributing to confusion and delayed follow-up. Government initiatives designed to simplify dementia care, such as informational gateway systems, were perceived as confusing and inadequate. Administrative burdens, including extensive paperwork, compounded caregiver stress during already challenging periods. Despite frequent hospitalizations, access to palliative care consultations remained limited, creating uncertainty for families.<br><br>**1.3 Medication management across transitions:**<br>Inconsistent medication reconciliation, poor communication between care providers, and limited cognitive assessments contributed to inappropriate prescribing and medication errors. Caregivers reported both over- and under-medication, particularly with sedatives that impaired alertness. Polypharmacy and inadequate discharge counselling increased the risk of adverse drug events and	reduced uncertainty. High-quality, lay-friendly discharge documentation improved understanding of care plans, while documented end-of-life preferences reduced stress for caregivers.<br><br>**1.3 Medication management across transitions:**<br>Transitions between care settings provided key opportunities for medication review and optimization. Interdisciplinary collaboration, especially involving pharmacists, improved medication management and ensured tailored medication lists were provided. Structured medication education, including detailed discharge counselling and follow-up guidance, supported caregivers in safe administration. Effective communication among healthcare providers during transitions enhanced continuity and safety.<br><br>**1.4 Financial and policy constraints:**<br>Supportive policy environments that subsidize transitional and

			hospital readmissions. People with dementia were less likely to have accurate medication histories recorded, resulting in gaps in care continuity.<br><br>**1.4 Financial and policy constraints:** Financial limitations, restrictive payer systems, and a lack of subsidized services reduce available care options and place significant financial strain on caregivers and families. These structural inequities often determine whether individuals can access quality transitional care.<br><br>**1.5 Structural and system-level facilitators and innovations supporting transitions:**<br>Caregivers frequently report feeling exploited, perceiving that healthcare systems depend excessively on unpaid family members to sustain care. This overreliance on informal caregivers reflects broader structural deficiencies, such as workforce shortages and inadequate community-based support.	community-based services reduce the financial burden on caregivers. Simplified funding mechanisms and equitable access to financial assistance enable families to make timely and appropriate care decisions.<br><br>**1.5 Structural and system-level facilitators and innovations supporting transitions:**<br>Comprehensive, person-centered models of care—such as home-based primary care, care coordination programs, and Admiral Nurse services, help ensure continuity and reduce caregiver burden. Proactive advance care planning and early palliative discussions align care with the individual's and family's preferences. Ongoing relationships with primary care providers build trust and promote early identification of support needs. Telehealth, transport assistance, and community referral pathways improve access and help prevent avoidable care transitions.

**The role of the health and social care workforce in transitions**	32	(Armstrong et al., 2020)<br>(Ashbourne et al., 2021)<br>(Broadbent & Gilbert, 2020)<br>(Brooks et al., 2022a)<br>(Brooks et al., 2022b)<br>(de Vries et al., 2019)<br>(Cronfalk et al., 2018)<br>(Eyles et al., 2021)<br>(Fitzpatrick & Grace, 2019)<br>(Freedman et al., 2022)<br>(Gilmore-Bykovskyi et al., 2021)<br>(Gresham et al., 2018)<br>(Groenvynck et al., 2022)<br>(Harkin et al., 2020)<br>(Hui et al., 2022)<br>(Holton et al., 2023)<br>(Johannessen et al., 2019)<br>(Khemai et al., 2022)<br>(Manis et al., 2021)<br>(Mogan et al., 2022)<br>(Nguyen et al., 2022)<br>(Radcliffe et al., 2023)<br>(Rapp et al., 2018)<br>(Rognstad et al., 2020)<br>(Rubinsztein et al., 2020)<br>(Saragosa et al., 2023)<br>(Smith & Phillipson, 2022)<br>(Smith et al., 2023)<br>(Statz et al., 2022)	**2.1 Workforce knowledge and dementia-specific expertise:**<br>Lack of dementia-specific training and expertise across health and social care settings limited staff confidence and competence during transitions. Caregivers reported that staff often lacked understanding of dementia progression and subtype variations, particularly for behavioral symptoms associated with frontotemporal dementia (FTD) and dementia with Lewy bodies (DLB), leading to inappropriate placement decisions and frequent relocations. Limited palliative and end-of-life care expertise further hindered timely and compassionate discussions about care options.<br><br>**2.2 Empathy, communication, and staff attitudes**:<br>Healthcare staff sometimes failed to recognize how individuals with dementia perceive and respond to their environment, leading to miscommunication or distress. Poor, inconsistent, or overly technical communication left caregivers	**2.1 Workforce knowledge and dementia-specific expertise:**<br>Access to high-quality dementia education programs and frameworks, such as the Gold Standards Framework, improved staff confidence and care quality during transitions. Structured training in dementia-specific and end-of-life care enabled staff to better anticipate and manage the needs of people with dementia and their caregivers.<br><br>**2.2 Empathy, communication, and staff attitudes:**<br>Empathetic, compassionate staff who maintained respectful communication facilitated smoother transitions and emotional reassurance for families. Healthcare professionals who served as consistent, trusted contacts, sometimes referred to by caregivers as "cheerleaders, helped guide families through the transition process. Regular communication via phone, email, or in-person meetings,

		(Tropea et al., 2022)<br>(Wang et al., 2022b)<br>(Wright et al., 2023)<br>(Zmora et al., 2021)	feeling excluded from decision-making. Time constraints and workload pressures often prevented meaningful engagement or explanation of care plans.<br><br>**2.3 Staffing pressures and organizational constraints**:<br>Chronic staff shortages, high turnover, and limited protected time undermined continuity of care and disrupted relationship-building with families. Workplace pressures, including heavy caseloads and time demands, reduced opportunities for compassionate communication and individualized support. These organizational factors led to fragmented transitions and increased caregiver anxiety.<br><br>**2.4 Finding and transitioning into appropriate care settings:**<br>Caregivers found the process of locating suitable care facilities overwhelming and time-consuming, often compounded by long waiting lists and limited availability. The pressure to accept the first available	particularly from nurses and psychologists, helped families stay informed and supported.<br><br>**2.3 Staffing pressures and organizational constraints**:<br>Investment in workforce stability, adequate staffing levels, and protected time for staff to engage with families strengthened care continuity. Interdisciplinary teamwork and consistent staff assignments helped build trust, improving the coordination and quality of transitions.<br><br>**2.4 Finding and transitioning into appropriate care settings:**<br>The involvement of care home brokers or aged care placement consultants reduced caregiver stress by helping families navigate complex placement processes. Purpose-built memory support units and cottage-style respite facilities promoted resident wellbeing and a sense of familiarity. Personalized care approaches, such as maintaining

			bed created feelings of loss of control. Intermediate and respite care options were scarce, and attempts to arrange these services often left caregivers exhausted. Concerns about care quality, depersonalized routines, and unsafe environments, including risks of neglect or falls, were frequently raised.	routines, incorporating personal histories, and including familiar belongings, helped preserve identity and ease transitions for people living with dementia.
**Caregiving, emotions, and decision-making in transitions**	37	(Ashbourne et al., 2021)<br>(Brooks et al., 2022a)<br>(Cottrell et al., 2020)<br>(Cronfalk et al., 2018)<br>(Damien et al., 2020)<br>(Davison et al., 2019)<br>(de Vries et al., 2019)<br>(Fekonja et al., 2021)<br>(Fitzpatrick & Grace, 2019)<br>(Gilmore-Bykovskyi et al., 2021)<br>(Gresham et al., 2018)<br>(Groenvynck et al., 2022)<br>(Hanssen & Tran, 2019)<br>(Hähnel et al., 2023)<br>(Hanson et al., 2019)<br>(Hanssen et al., 2022)<br>(Harrison et al., 2023)<br>(Holton et al., 2023)	**3.1 Knowledge and support networks**<br>**Lack of knowledge:** Caregivers frequently reported limited understanding of dementia and available services, making it difficult to make informed transition decisions. Caregivers often also lacked confidence in their knowledge, leading to self-doubt about whether their expectations and staff actions were appropriate. The absence of transitional counselling and informal peer networks (e.g., buddy programs) left caregivers feeling unprepared. Inconsistent or non-existent support from family and friends	**3.1 Knowledge and support networks**<br>**Respite services:**<br>Access to respite care and educational resources reduced caregiver distress and delayed transitions to long-term care. Learning from peers and engaging with supportive networks enhanced caregivers' confidence in managing transitions. Receiving validation for their decisions strengthened caregivers' confidence and reduced guilt.<br>Reflective practices, such as journaling, helped caregivers reaffirm the necessity of care

		(Hui et al., 2022)<br>(Johannessen et al., 2019)<br>(Khemai et al., 2022)<br>(Leggett et al., 2018)<br>(Larsen et al., 2020)<br>(Mogan et al., 2022)<br>(Nguyen et al., 2022)<br>(Parker et al., 2021)<br>(Radcliffe et al., 2023)<br>(Rognstad et al., 2020)<br>(Rubinsztein et al., 2020)<br>(Saragosa et al., 2023)<br>(Sawan et al., 2021)<br>(Smith & Phillipson, 2022)<br>(Statz et al., 2022)<br>(Toles et al., 2022a)<br>(van der Heide et al., 2021)<br>(Wright et al., 2023)<br>(Zmora et al., 2021)	intensified feelings of grief, guilt, and isolation.<br>Without guidance or validation, caregivers experienced heightened emotional strain and confusion during transitions.<br><br>**3.2 Caregiver advocacy and recognition**<br>Caregivers frequently felt unacknowledged by healthcare professionals, leading to exclusion from discussions and decisions about care transitions.<br>Poor coordination and inconsistent information sharing between caregivers and healthcare providers caused disputes and mistrust.<br>Caregivers felt obligated to remain constantly alert to ensure quality care, which increased fatigue and emotional exhaustion. Some caregivers found it difficult to raise concerns or advocate for changes in care, especially within institutional settings.<br>Long wait times and the need to repeatedly assert themselves	transitions and process emotional responses.<br><br>**3.2 Caregiver advocacy and recognition**<br>**Continued involvement:** Ongoing participation in the person's care allowed caregivers to maintain routines and ensure continuity. Active advocacy by caregivers ensured that the needs of people with dementia were recognized and addressed. Caregivers' presence during hospital stays was perceived as calming and supportive for their loved ones. Collaborative participation in care planning, medication management, and discharge processes contributed to personalized, effective care. Assertive yet respectful caregiver involvement was associated with smoother transitions and better outcomes for people living with dementia.<br><br>**3.3 Emotions about timing and decision-making**

			during acute episodes added stress and frustration.<br><br>**3.3 Emotions about timing and decision-making**<br>**Uncertainty in planning:** Families often faced uncertainty about when and how to prepare for increasing care needs as dementia progressed. Unplanned or hasty transitions created distress and disrupted continuity of care for both caregivers and individuals with dementia.<br>Delays in decision-making often led to sudden admissions to long-term care, exacerbating emotional strain. Caregivers experienced guilt and regret about transitioning too late or too early, feeling conflicted about timing decisions.<br>Differences in emotional readiness between caregivers and individuals with dementia complicated proactive decision-making.<br><br>**3.4 Emotional adjustment, conflict, and identity:**	**Timely diagnosis:** Early diagnosis and prognosis discussions helped families anticipate future care needs and plan transitions proactively.<br>Allowing families adequate time to prepare and visit potential facilities reduced stress and improved decision-making. Clear guidance, timelines, and defined responsibilities helped caregivers navigate transition planning more effectively. Families who initiated transitions before a crisis reported greater control and less regret. Structured care plans involving all stakeholders and encouraging early discussion of future needs led to smoother transitions.<br><br>**3.4 Emotional adjustment, conflict, and identity**<br>**Joint decision-making:** Transitions involving both the caregiver and the person with dementia in decision-making improved acceptance and emotional adjustment. Many

			Both caregivers and individuals living with dementia often experienced reluctance, anxiety, and stress about transitions, which complicated decision-making. Transitions frequently triggered conflict between caregivers and individuals with dementia, sometimes escalating to crisis situations requiring urgent intervention. Caregivers described a loss of control over care decisions, particularly when healthcare professionals made final determinations. Many caregivers experienced persistent grief, loneliness, and a loss of personal identity following the transition, particularly spouses. Feelings of guilt, sadness, and anger were common, especially when transitions occurred under crisis circumstances or against family wishes.	families reported feelings of relief and reassurance once settled into care, noting improvements in safety and wellbeing. Long-term care placements often enhanced social engagement and quality of life for individuals compared to remaining at home. Over time, caregivers often came to accept the transition, feeling they had made the best possible decision under difficult circumstances. Caregivers who arranged transitions proactively reported less guilt and emotional distress than those who waited until a crisis occurred.
**Cultural, social, and situational influences on transition pathways**	9	(de Vries et al., 2019)<br>(Fekonja et al., 2021)<br>(Hanssen & Tran, 2019)<br>(Hanssen et al., 2022)	Caregivers often experienced strong feelings of guilt, shame, or failure when transitioning a loved one with dementia into long-term care,	Aligning services with cultural values, beliefs, and caregiving norms helped families view professional care as compatible

		(Holton et al., 2023)<br>(Hui et al., 2022)<br>(Johannessen et al., 2019)<br>(Pond et al., 2019)<br>(Statz et al., 2022)	particularly when influenced by the opinions or disapproval of family and friends.<br><br>In collectivist and family-oriented cultures, moral expectations to care for older relatives at home created resistance to seeking professional or residential care.<br><br>Fear of judgment from extended family, community members, or society at large contributed to hesitation and emotional conflict around care transitions. These social pressures reinforced the perception that institutional care signified personal or familial failure.	with their cultural expectations.<br><br>Normalizing the use of professional and residential care, alongside counselling or peer support, reduced feelings of guilt and self-blame.<br><br>Addressing stigma: Education and culturally sensitive outreach programs that promoted positive narratives about dementia care reduced stigma and improved acceptance of formal support.
